# Energetic Di- and Trinitromethylpyridines: Synthesis and Characterization

**DOI:** 10.3390/molecules23010002

**Published:** 2017-12-21

**Authors:** Yiying Zhang, Xiaoyu Sun, Shannan Yu, Lingxiang Bao, Chenghui Sun, Siping Pang

**Affiliations:** School of Material Science & Engineering, Beijing Institute of Technology, Beijing 100081, China; zhangyy989312@126.com (Y.Z.); sxy1204254800@126.com (X.S.); nandyu@163.com (S.Y.); blx823@126.com (L.B.)

**Keywords:** energetic materials, trinitromethyl, dinitromethyl, pyridine, detonation properties

## Abstract

Pyridine derivatives based on the addition of trinitromethyl functional groups were synthesized by the reaction of N_2_O_4_ with the corresponding pyridinecarboxaldoximes, then they were converted into dinitromethylide hydrazinium salts. These energetic compounds were fully characterized by IR and NMR spectroscopy, elemental analysis, differential scanning calorimetry (DSC), and X-ray crystallography. These pyridine derivatives have good densities, positive enthalpies of formation, and acceptable sensitivity values. Theoretical calculations carried out using Gaussian 03 and EXPLO5 programs demonstrated good to excellent detonation velocities and pressures. Each of these compounds is superior in performance to TNT, while 2,6-bis(trinitromethyl)pyridine (D = 8700 m·s^−1^, P = 33.2 GPa) shows comparable detonation performance to that of RDX, but its thermal stability is too low, making it inferior to RDX.

## 1. Introduction

There are currently ongoing efforts to synthesize novel energetic materials with improved performance and decreased sensitivity [1,2,3,4,5,6]. However, these requirements are quite often mutually exclusive, such that the desired combination of high detonation performance with maximum possible chemical stability is a major challenge. Over the last few decades, several strategies for the design of energetic materials that have a high energy content in conjunction with good thermal and mechanical stabilities have been developed [7,8], one of which is to replace benzene rings with nitrogen-containing heterocycles as fundamental components of molecular structures [2,9]. Compared with the benzene ring, nitrogen-containing heterocycles typically possess a greater enthalpy of formation as well as a higher density and more positive oxygen balance [10,11], all of which are desirable characteristics for new energetic compounds. In recent years, the application of azine as a new energetic skeleton has attracted considerable interest, and many polynitro-azine derivatives have been designed and synthesized [12]. However, due to the electron deficient character of the azine ring, it is difficult to perform electrophilic aromatic substitution on the ring, so the introduction of nitro groups into azine rings remains a challenging task in practice.

Nitro-based moieties represent the most common energetic functional group, and contribute markedly to the overall performance of an energetic compound by enhancing both the oxygen balance (OB) and density [13,14,15,16,17]. Among these groups, the trinitromethyl group has the highest oxygen content, and its introduction to a molecule is essentially equivalent to adding two nitro groups (since one of the nitro groups is necessary for the complete oxidation of the carbon atom in the methyl group). The incorporation of trinitromethyl moieties into an azine ring is thus a potential means of increasing the OB so as to improve the detonation performance, and some trinitromethylazines have been synthesized using various methods. As an example, 2,4,6-tris(trinitromethyl)-1,3,5-triazine was obtained by the destructive nitration of the carboxyl groups to trinitromethyl entities in 2,4,6-tris(dicarboxymethylene)-1,3,5-triazine by action of concentrated HNO_3_ [18]. This compound is an oxidant and has a high density (2.00 g/cm^3^), but it is unstable in the air and very easy to hydrolyze. Trinitromethyl-substituted pyrazine was also obtained as an unexpected product by the treatment of 2-alkoxy-3-methylpyrazine with an HNO_3_/H_2_SO_4_ mixture [19]. The attachment of trinitromethyl groups with a pyridine ring has also been attempted by different techniques. Gakh and Khutoretskii reported the synthesis of 2-trinitromethylpyridine by the reaction of *N*-fluoropyridinium fluoride with trinitromethane [20]. Pyridine with methyl groups in the 2, 3, and 4 positions behaved in the same way and the corresponding trinitromethyl-substituted methylpyridines were obtained. Subsequently, they also studied the behavior of this series of trinitromethylpyridines upon electron impact [21]. Another synthesis method for 2-trinitromethylpyridine was reported in 1993 by the ozone-mediated *C*-nitration of methylpyridines with nitrogen dioxide [22]. More recently, Katritzky et al. obtained 5-methyl-2-trinitromethylpyridine on the nitration of 2,5-lutidine with nitric acid in trifluoroacetic anhydride [23]. However, these methods shared an obvious disadvantage in that the yields were rather low (below 20%).

**Figure 1:**
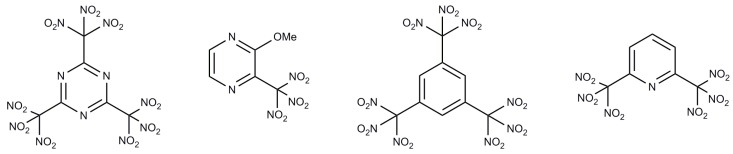
Representative trinitromethylazines/trinitromethylbenzenes and bis(trinitromethyl)-substituted pyridine.

In contrast, the synthesis route of 2-trinitromethylpyridine through the reaction of N_2_O_4_ with pyridine-2-carboxaldoxime, which was developed at the N.D. Zelinsky Institute of Organic Chemistry, appeared to be a favorable method for high yield (more than 60%) [24]. In fact, a series of trinitromethyl-substituted arenes and hetarenes have been prepared through the interaction of N_2_O_4_ with corresponding carboxaldehyde oximes [25,26]. Although tris(trinitromethyl)benzene has been synthesized by the reaction [25], to the best of our knowledge, heterocyclic compounds with more than one trinitromethyl group synthesized in this way have not yet been reported. Hence, introducing more trinitromethyl groups into the pyridine ring becomes an attractive but challenging objective, as it requires overcoming electronic and stereo-hindrance effects (Figure 1). At the same time, in consideration of the well-known instability of the trinitromethyl moiety at higher temperatures, the ionization of trinitromethyl-substituted pyridines is also worth exploring, as ionic compounds usually display better stabilities than their nonionic precursors [27]. An interesting denitration reaction of trinitromethyl groups into dinitromethyl groups in heterocycle substrates has been studied. For example, The denitration of 2-trinitromethylpyridine with potassium iodide in methanol allowed for the obtainment of the potassium salt of 2-(dinitromethyl)pyridine [24]. The reaction of mono(trinitromethyl)-1,3,5-triazines with potassium iodide in methanol resulted in dinitromethyl-1,3,5-triazine potassium salts [28]. Additionally, the conversion of trinitromethyl groups attached to imidazoles, triazoles, and tetrazoles into the corresponding dinitromethylide salts following treatment with hydrazine has been reported [4,29,30]. Based on these successful examples, the trinitromethyl-substituted pyridines in our research were subject to attempted denitration using hydrazine.

In our continuing search for new high-performance polynitro energetic materials, we are interested in developing novel trinitromethyl-substituted heterocyclic compounds. Thus, in the present study, we attempted to introduce more trinitromethyl groups onto the pyridine ring to increase energetic performance. These trinitromethyl-substituted pyridines were subsequently converted into dinitromethylide hydrazinium salts. Each of these compounds was fully characterized and their energetic properties were determined either empirically or theoretically.

## 2. Results and Discussion

### 2.1. Syntheses

**Scheme 1:**
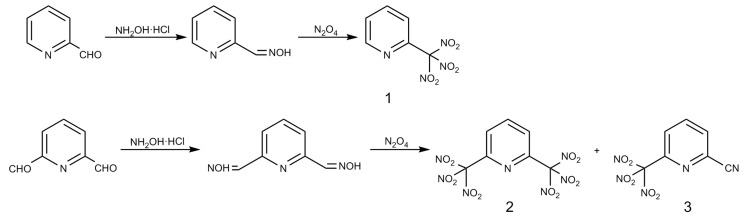
The synthetic routes of **1**–**3**.

**Scheme 2:**
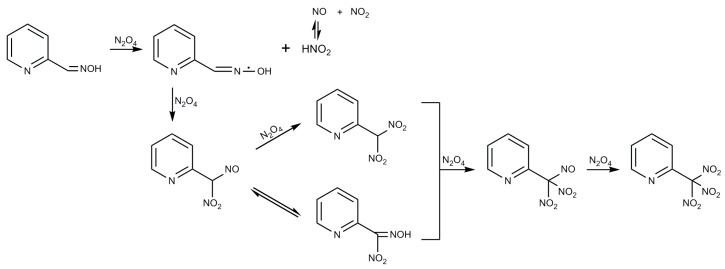
The reaction mechanism for the synthesis of 2-trinitromethylpyridine.

**Scheme 3:**
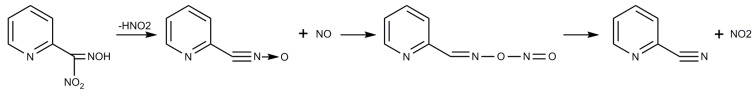
The reaction mechanism for the formation of the cyano-compound.

As was stated in the introduction, 2-trinitromethylpyridine (**1**) was synthesized through the reaction of N_2_O_4_ with pyridine-2-carboxaldoxime (Scheme 1). Based on a method reported in literature, a simpler procedure was employed. N_2_O_4_ was added into a solution of pyridine-2-carboxaldoxime at room temperature and the purification of products was carried out by column chromatography in a Biotage Isolera One apparatus. The reaction mechanism has been given in the literature earlier (Scheme 2) [25,31]. The preparation of bis(trinitromethyl)-substituted pyridine was somewhat more challenging. Firstly, compared with the preparation of 1, the reaction of pyridine-2,6-dicarboxaldehyde dioxime with N_2_O_4_ required a higher reaction temperature (60 °C rather than 40 °C). In addition, X-ray diffraction (XRD) data indicated that the reaction yielded two main products: the desired 2,6-bis(trinitromethyl)pyridine (**2**) and the byproduct 2-cyano-6-(trinitromethyl)pyridine (**3**). It was found that increasing the N_2_O_4_ concentration (within the range of 0.5‒2.5 mL N_2_O_4_/mmol pyridine-2,6-dicarboxaldehyde dioxime) increased the relative proportion of 2 in the product mixture. The formation of the by-product 3 is also explained based on literature reports (Scheme 3) [32]. Firstly, the intermediate nitrolic acid underwent the loss of a molecule of nitrous acid and the nitrile oxide was obtained. In the second step, nitrogen monoxide removed an oxygen atom with the formation of nitrile and nitrogen dioxide.

**Scheme 4:**
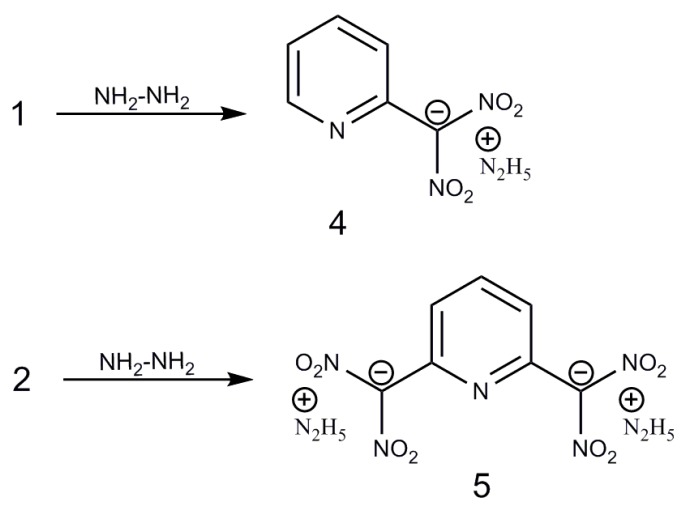
The syntheses of dinitromethylpyridine salts **4** and **5**.

Following the preparation of the above compounds, the ionization of 1 and 2 was attempted. Based on reports, the analogous denitration of trinitromethyl-substituted pyridines using hydrazine was examined in our research. The trinitromethyl-substituted pyridine 1 was first reacted with 80% aqueous hydrazine, and the light yellow hydrazinium salt of 2-dinitromethylpyridine (**4**) was obtained in high yield (Scheme 4). Subsequently, 2,6-bis(trinitromethyl)pyridine was also successfully converted into the dihydrazinium salt of 2,6-bis(dinitromethyl)pyridine (**5**). To the best of our knowledge, this is the first report of the preparation of a dihydrazinium salt in this manner.

All the compounds prepared in this work were stable in air and could be stored for extended periods of time. Their structures were confirmed by infrared (IR) and nuclear magnetic resonance (NMR) spectroscopy, mass spectrometry (MS), and elemental analysis. Compounds **1**, **2**, **3**, **4** and **5** were also characterized by X-ray crystallography. Selected data and X-ray structural parameters are provided in the Extended Supplemental Information.

### 2.2. X-ray Crystallography

**Figure 2:**
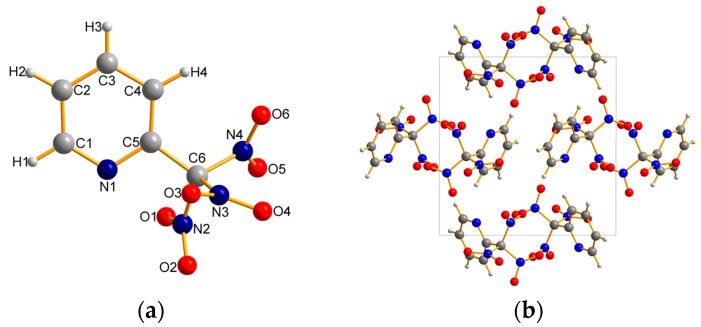
(**a**) Molecular structure of **1**; (**b**) packing diagram of 1 viewed down the a-axis.

**Figure 3:**
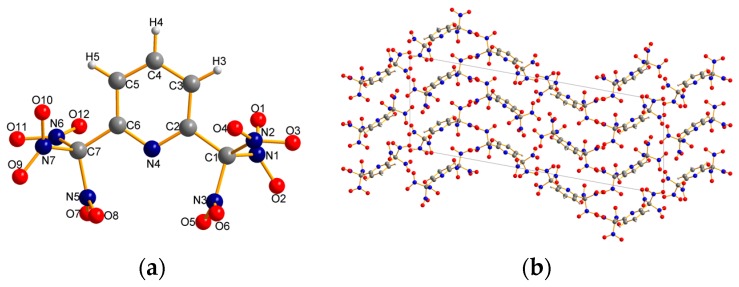
(**a**) Molecular structure of **2**; (**b**) packing diagram of 2 viewed down the a-axis.

**Figure 4:**
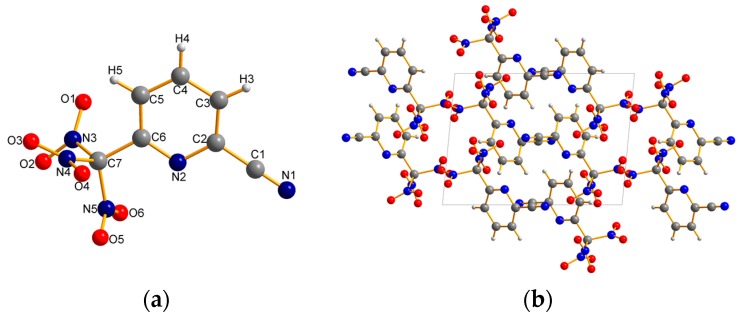
(**a**) Molecular structure of **3**; (**b**) packing diagram of 3 viewed down the b-axis.

**Figure 5:**
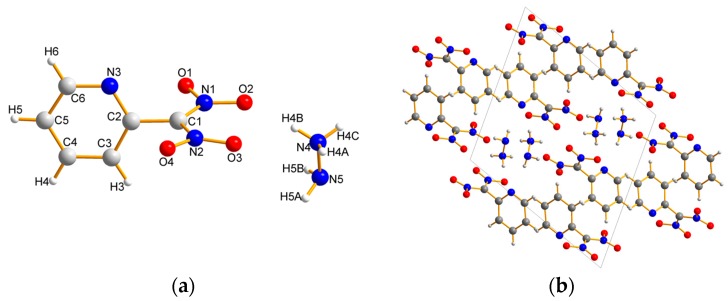
(**a**) Molecular structure of **4**; (**b**) packing diagram of 4 viewed down the b-axis.

**Figure 6:**
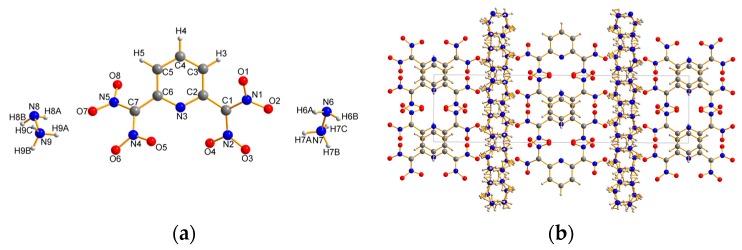
(**a**) Molecular structure of **5**. The disordered hydrazinium cations were partly deleted for clarity; (**b**) packing diagram of 5 viewed down the c-axis.

Single crystals of **1**, **2** and **3** suitable for single-crystal XRD were obtained by the slow evaporation of dichloromethane and n-hexane solutions of these compounds at room temperature, whereas crystals of **4** and **5** were obtained from methanol and water, respectively. All crystals were stable at room temperature and were found not to be hygroscopic. Their molecular structures are shown in Figure 2, Figure 3, Figure 4, Figure 5 and Figure 6 and the crystallographic data are summarized in Table 1.

Compound **1** crystallizes in the monoclinic space group *P*2_1_/*c* with four molecules per unit cell and has a calculated density of 1.692 g·cm^−3^ at 153 K (Figure 2). The bond lengths and bond angles in the pyridine ring of this compound are in the typical ranges [33], although the C6–N2, C6–N3, and C6–N4 bond lengths in the trinitromethyl group are 1.528, 1.538, and 1.524 Å, respectively, all of which are longer than a standard C–N single bond (1.460 Å) [34]. The C6 atom is in the plane of the pyridine ring and is associated with the torsion angles C1–N1–C5–C6 = −179.47° and C3–C4–C5–C6 = −179.68°. The pyridine ring and three nitro groups are attached to C6 in a tetragonal configuration.

Compound **2** crystallizes in the triclinic space group *P*$\bar{1}$ with eight molecules per unit cell and a calculated density of 1.832 g·cm^−3^ at 105 K (Figure 3). Compared to **1**, its density is significantly increased because of the introduction of the second trinitromethyl group. Similar to **1**, the C–N bond lengths in the trinitromethyl groups are longer than normal C–N single bonds. The large volume of the two trinitromethyl groups makes the region beside the pyridine N atom quite crowded and they stretch in an optimal conformation, trying to decrease the repulsive strain. The N1, N2, N6 and N7 atoms are distributed in an approximately symmetric manner on both sides of the pyridine ring. The N5 atom and the pyridine ring are almost coplanar, as can be seen from the N4–C6–C7–N5 torsion angle (−3.65°), while the N3 is slightly twisted out of this plane based on the N4–C2–C1–N3 torsion angle (−15.7°). However, it is evident that a repulsive strain is still present, as demonstrated by a comparison of bond angles; the N4–C2–C1 and N4–C6–C7 bond angles are 114.7° and 113.8°, respectively, while the corresponding bond angle in compound 1 is 111.9°.

Compound **4** crystallizes in a monoclinic crystal system with space group *P*2_1_/*n* and a density of 1.617 g·cm^−3^ at 153 K (Figure 5). The C–N and C–C bond lengths of the pyridine ring are in the expected ranges. Compared to **1**, the C1–C2 bond length (1.484 Å) is slightly shorter. In addition, the C1–N1 and C1–N2 bond lengths of the dinitromethyl group are 1.362 Å and 1.391 Å, respectively, and thus are both much shorter than the analogous bonds in 1 and normal C–N single bonds. This can be explained by the change in the electron density distribution after the removal of a nitro substituent of the trinitromethyl group. Compared with the earlier reported crystal structures of several zwitterionic dinitromethylazine derivatives in which the protonation occurred at one of the nitrogens of the azine ring [35,36], the only nitrogen atom in the pyridine ring was not protonated, while the N_2_H_5_^+^ cation was formed, which was the same situation with the analogical reactions in earlier literature [4,29,30]. Interestingly, the remaining atoms of the dinitromethyl group are inclined to form a plane with the torsion angles O4–N2–C1–N1 = 171.24° and O2–N1–C1–N2 = 3.7°. The two planes defined by the dinitromethyl group and the pyridine ring are almost perpendicular (N3–C2–C1–N2 = 94.53° and C3–C2–C1–N1 = 95.42°).

Compound **5** crystallizes in an orthorhombic crystal systemspace group *Pbcn* with a calculated density of 1.782 g·cm^−3^ at 153 K and four molecules in a unit cell (Figure 6). Disorder of hydrazinium cations was observed in the crystal. Similar to compound **4**, the bonds connecting the pyridine and dinitromethyl groups as well as the C–NO_2_ bonds become shorter than its parent compound **2** and the protonation sites are also located in the nitrogen atoms of hydrazine. However, the remaining atoms of each dinitromethyl group in compound **5** are not inclined to be coplanar with the torsion angles (O1–N1–C1–N2 and O8–N5–C7–N5 = −160.70°; O3–N2–C1–N1 and O6–N4–C7–N5 = 9.07°), which is different from compound **4**.

### 2.3. Physicochemical and Energetic Properties

The physicochemical and energetic properties of each of the compounds are summarized in Table 2.

In this work, the thermal stabilities of all compounds were determined by differential scanning calorimetry (DSC) measurements scanning at 5 °C·min^−1^ under dry, oxygen-free nitrogen over the temperature range 25–500 °C. The melting points of compounds **1**‒**3** were in the range 68–96 °C, while their decomposition temperatures ranged from 101 to 114 °C. Both **1** and **3** were found to have lower melting points than TNT. These two compounds were also observed to have very similar melting points, but the latter had a significantly lower decomposition point, demonstrating that the introduction of a cyano group has negative effects on the thermal behavior of **1**. The introduction of the second trinitromethyl in the case of **2** significantly increases the melting temperature, but its thermal stability becomes worse with a decomposition temperature of 101 °C. The relatively low decomposition points of **1**, **2** and **3** can be attributed to the well-known instability of the trinitromethyl group at higher temperatures. However, when the trinitromethyl group underwent the loss of a nitro group and was converted to a dinitromethylide salt, salts **4** and **5** displayed much better thermal stability than their parent compounds, with decomposition temperatures of 181 and 125 °C, respectively.

The sensitivities of these compounds towards impact (IS) and friction (FS) were determined according to the standard BAM methods [39]. Compound **1** (IS = 16 J, FS > 360 N) has impact and friction sensitivities similar to those of TNT. As a result of the introduction of the second trinitromethyl group, 2 (IS = 9 J, FS = 192 N) is more sensitive than 1 but still less sensitive than RDX (IS = 7.4 J, FS = 120 N). As noted, the formation of salts is often an effective means of reducing sensitivity [40]. However, salt 4 possesses similar sensitivity to its parent compound **1**, and is even slightly more sensitive regarding impact sensitivity. In contrast, salt **5** is significantly less sensitive than **2**, especially with regard to FS.

OB plays an important role in determining the detonation properties of energetic compounds. Comparing the OB values of **1**‒**5**, **2** has the highest value of −14.85%, exceeding that of 2,4,6-trinitropyridine-1-oxide (TNPyO), which has the best OB (−27.8%) among the previously-reported energetic pyridine derivatives. The OB values of the hydrazinium salts are lower due to the addition of cations and the loss of nitro groups, although salt **5** still has a good OB comparable to that of **1**.

Density is another important physical property of energetic materials. The densities of all the compounds were measured using a gas pycnometer at 25 °C, and were found to be in the range of 1.56 (**4**)–1.77 g·cm^−3^ (**2**). Although the bulky trinitromethyl group usually has a negative effect on crystal packing, the densities of these compounds increase in proportion to the accumulation of trinitromethyl groups, such that **2** possesses a significantly improved density compared to **1**. The enthalpies of formation were computed based on the Gaussian 03 program package [41] and each compound exhibited a positive enthalpy of formation. Interestingly, the byproduct **3** had the highest value (314.22 kJ·mol^−1^), most likely due to the high bond energy of the cyanogroup. Based on the calculated enthalpies of formation and the measured densities at ambient temperature, the detonation properties of all the compounds were determined using the EXPLO5 (v6.01) program [42]. The calculated detonation velocities (D) and detonation pressures (P) were in the range of 7438–8700 m·s^−1^ and 19.6–33.2 GPa, respectively. Compound **1** exceeds TNT in terms of detonation properties, while **2** exhibits superior performance to **1**. The performance of the former also exceeds that of presently-known energetic pyridine derivatives and is only slightly lower than that of RDX. The detonation properties of salts **4** and **5** are lower than those of their parent compounds.

In conclusion, compound **2** possesses superior detonation properties and acceptable sensitivity values comparable to those of RDX, but its thermal stability is too low for practical applications. Through the denitration reaction and formation of hydrazinium salts, compounds **4** and **5** display significantly improved thermal stability.

## 3. Materials and Methods

Caution

Although none of the compounds described herein exploded or detonated in the course of this research, these energetic materials should be handled with extreme care using best safety practices (including the use of personal protective equipment such as leather gloves, face shields, and ear plugs).

### 3.1. General Information

All starting materials were commercially available and were used as-received. IR spectra were obtained from KBr pellets using a Nicolet Magna IR 560 spectrophotometer (Madison, WI, USA) over the range of 400–4000 cm^−1^. ^1^H-NMR and ^13^C-NMR spectra were recorded on a Bruker ARX-400 instrument (Zurich, Switzerland). Chemical shifts are reported in ppm relative to tetramethylsilane. Elemental analyses (C, H and N) were performed using an Elementar Vario EL (Bremen, Germany). Liquid chromatography (LC)-MS electrospray ionization (ESI) data were acquired with an Agilent 6120 LC-MS spectrometer (Santa Clara, CA, USA). Crystal structures were determined on a Rigaku RAXIS IP diffractometer (Rigaku Corporation, Tokyo, Japan) with the SHELXTL crystallographic software package of molecular structure. CCDC 1,582,435, 1,583,118, 1,582,533, 1,582,458 and 1,583,040 contain the supplementary crystallographic data for this paper. These data can be obtained free of charge from The Cambridge Crystallographic Data Centre via http://www.ccdc.cam.ac.uk/data_request/cif. The melting and decomposition points of the compounds were determined using a TA-DSC Q2000 differential scanning calorimeter (New Castle, DE, USA) at a scanning rate of 10 °C min^−1^. Densities were measured at 25 °C using a Micromeritics Accupyc II 1340 gaspycnometer (Norcross, GA, USA). The impact and friction sensitivity measurements were carried out with a BAM fall hammer apparatus (BFH-10) and a BAM friction apparatus (FSKM-10) (San Diego, CA, USA), respectively.

### 3.2. Theoretical Studies

Calculations were performed using the Gaussian 03 suite of programs [41]. The geometric optimization of the structures and frequency analyses employed the density functional theory (DFT) B3LYP method with the 6-311+G** basis set and single-point energies were calculated at the MP2/6-311+G** level. Each optimized structure was characterized to determine the true local energy minima on the potential energy surface without imaginary frequencies. The heats of formation (∆H_f_) of the neutral molecules and the corresponding anions were computed using the isodesmic reaction and protonation reaction [43,44]. The enthalpy of each isodesmic reaction was obtained by combining the MP2/6-311+G** energy difference for the reaction, the scaled zero-point energies (B3LYP/6-311+G**), and other thermal factors (B3LYP/6-311+G**). Thus, the heats of formation of the cations and anions being investigated could be readily extracted. Finally, the heat of formation of each salt was obtained by combining the enthalpies of the cation and anion with the lattice energy of the salt according to the Born-Haber cycle. Using these heats of formation and densities, detonation velocities and detonation pressures were calculated using the EXPLO5 v6.01 program (Brodarski Institute, Zagreb, Croatia) according to the Kamlet-Jacobs equations [45,46,47].

### 3.3. Experiment Procedures

*2-trinitromethylpyridine* (**1**): Pyridine-2-carboxaldoxime (488 mg, 4 mmol) was dissolved in acetonitrile (20 mL) at room temperature, after which N_2_O_4_ (5 mL) was added dropwise with vigorous stirring. The reaction mixture was subsequently heated to 40 °C and maintained at this temperature for 2 h. Then the mixture was cooled to room temperature and stirred vigorously to remove residual N_2_O_4_. The solvent was evaporated under vacuum and the residue was purified by column chromatography in a Biotage Isolera One apparatus using a Flash Silica-CS column (25 g) and eluting with n-hexane and dichloromethane. The product **1** was isolated with satisfactory purity as a white solid (620 mg, 68%).

R_f_ = 0.7 (silica gel, 33% EtOAc/hexanes); T_d_ = 114 °C; ^1^H-NMR (CDCl_3_): δ 8.76 (d, *J* = 4.7 Hz, 1H), 7.96 (m, 2H), 7.62 (m, 1H); ^13^C-NMR (CDCl_3_): δ 150.32 (s), 142.72 (s), 138.09 (s), 128.10 (s), 125.19 (s); MS (ESI) *m/z*: 227 [M − 1]^−^; The other characterization data including the melting point, IR spectra, and elemental analysis were consistent with the literature report [24].

*2,6-bis(trinitromethyl)pyridine* (**2**) and *2-cyano-6-trinitromethylpyridine* (**3**): N_2_O_4_ (10 mL) was added dropwise to a suspension of pyridine-2,6-dicarboxaldehyde dioxime (660 mg, 4 mmol) in acetonitrile (40 mL) with vigorous stirring. The solid was observed to gradually dissolve and the reaction mixture eventually became dark brown. The mixture was subsequently heated to 60 °C and stirred at this temperature for 2 h, then cooled to room temperature and stirred vigorously to remove any residual N_2_O_4_. The solvent was evaporated under vacuum and the residue was purified by column chromatography, using a Biotage Isolera One apparatus (Uppsala, Sweden) with a Flash Silica-CS column (25 g), eluting with n-hexane and dichloromethane. Both **2** and **3** were isolated with satisfactory purity as a white solid (536 mg, 35.5%) and an orange solid (148 mg, 14.6%), respectively.

**Compound 2**: R_f_ = 0.5 (silica gel, 33% EtOAc/hexanes); m.p. 96–98 °C; T_d_ = 101 °C; ^1^H-NMR (CDCl_3_): δ 8.43 (s, 1H); ^13^C-NMR(CDCl_3_): δ 142.78 (s), 140.78 (s), 129.55 (s); IR (KBr): 3095, 2922, 2853, 1622, 1589, 1450, 1349, 1291, 1170, 1078, 999, 856, 804, 785 cm^−1^; MS (ESI) *m*/*z*: 376 [M-1]^−^, 331 [M-46]^−^; Elemental analysis for C_7_H_3_N_7_O_12_: calculated C 22.29, H 0.80, N 26.00%; measured C 22.21, H 1.01, N 25.68%.

**Compound 3**: R_f_ = 0.4 (silica gel, 33% EtOAc/hexanes); m.p. 68–72 °C; T_d_ = 106 °C; ^1^H-NMR (CDCl_3_): δ 8.25 (m, 2H), 8.06 (dd, *J* = 6.3, 2.4 Hz, 1H); ^13^C-NMR (CDCl_3_): δ 144.13 (s), 139.89 (s), 134.43 (s), 132.26 (s), 128.42 (s), 115.12 (s); IR (KBr): 3086, 2921, 1630, 1594, 1447, 1347, 1293, 1208, 1169, 1097, 1042, 993, 798, 735 cm^−1^; MS (ESI) *m/z*: 252 [M − 1]^−^, 207 [M − 46]^−^; Elemental analysis for C_7_H_3_N_5_O_6_: calculated C 33.21, H 1.19, N 27.67%; measured C 33.09, H 1.26, N 27.48%.

*Hydrazinium 2-dinitromethylpyridine* (**4**): An 80% solution of hydrazine hydrate (3 mmol) was added slowly to a solution of **1** (1 mmol) in methanol (5 mL). The resulting mixture was stirred at room temperature for 4 h, during which time a light-yellow precipitate was formed. The precipitate was filtered off, washed with cold ethanol, and recrystallized from methanol:water = 1:1 (*v*/*v*) to give the target product. (173 mg, 80.5%).

R_f_ = 0.7 (silica gel, 80% EtOAc/MeOH); T_d_ = 181 °C (decomposition without melting); ^1^H-NMR (DMSO-*d*_6_): δ 8.51 (d, *J* = 4.4 Hz, 1H), 7.77 (dd, *J* = 10.9, 4.4 Hz, 1H), 7.50 (d, *J* = 7.9 Hz, 1H), 7.18 (m, 1H); ^13^C-NMR (DMSO-*d*_6_): δ 151.70 (s), 148.54 (s), 136.39 (s), 134.20 (s), 125.26 (s), 121.62 (s); IR (KBr): 3333, 3125, 1587, 1489, 1462, 1376, 1329, 1303, 1243, 1128, 1091, 1054, 951, 797, 748, 696 cm^−1^; Elemental analysis for C_6_H_9_N_5_O_4_: calculated C 33.49, H 4.22, N 32.55%; measured C 33.32, H 4.31, N 32.62%.

*Dihydrazinium 2,6-bis(dinitromethyl)pyridine* (**5**): An 80% hydrazine hydrate solution (6 mmol) was added slowly to a solution of **2** (1 mmol) in methanol (5 mL). The resulting mixture was stirred at room temperature for 4 h, during which time a yellow precipitate was formed. The precipitate was filtered off, washed with cold ethanol, and recrystallized from methanol:water = 1:1(*v*/*v*) to give the target product. (267 mg, 76.1%).

R_f_ = 0.3 (silica gel, 33% EtOAc/MeOH); T_d_ = 125 °C (decomposition without melting); ^1^H-NMR (DMSO-*d*_6_): δ 7.71 (t, *J* = 7.5 Hz, 1H), 7.31 (d, *J* = 7.7 Hz, 2H); ^13^C-NMR (DMSO-*d*_6_): δ 151.30 (s), 136.43 (s), 134.60 (s), 123.32 (s); IR (KBr): 3362, 3113, 1586, 1491, 1447, 1415, 1335, 1278, 1224, 1171, 1128, 1096, 1070, 927, 815, 752, 720 cm^−1^; Elemental analysis for C_7_H_13_N_9_O_8_: calculated C 23.94, H 3.73, N 35.89%; measured C 23.85, H 3.59, N 36.02%.

## 4. Conclusions

The mono and bis(trinitromethyl)-substituted pyridines **1** and **2** were synthesized in moderate to excellent yields via the reaction of the corresponding pyridinecarboxaldoximes with N_2_O_4_. The by-product **3** was also separated and identified. Compounds **1** and **2** reacted with aqueous hydrazine to give dinitromethyl hydrazinium salts. All the compounds were fully characterized and their energetic properties were both measured and calculated. Due to the successful introduction of two trinitromethyl groups onto the pyridine ring, **2** possesses the best oxygen balance (−14.85%) and superior detonation properties (D = 8700 m·s^−1^, P = 33.2 GPa) compared to other energetic pyridine derivatives, in conjunction with moderate sensitivity values (IS = 9 J, FS = 192 N) comparable to those of RDX, but its thermal stability is too low for practical applications making it inferior to RDX. The formation of salt **5** significantly improves the thermal stability of **2** while maintaining much of the detonation performance.

## Figures and Tables

**Table 1:** Crystallographic data for **1**, **2**, **3**, **4** and **5**.

	1	2	3	4	5
CCDC	1,582,435	1,583,118	1,582,533	1,582,458	1,583,040
Empirical formula	C_6_H_4_N_4_O_6_	C_7_H_3_N_7_O_12_	C_7_H_3_N_5_O_6_	C_6_H_9_N_5_O_4_	C_7_H_13_N_9_O_8_
Formula mass	228.12	377.14	253.13	215.17	351.26
Temperature/K	153(2)	105	126	153(2)	153(2)
Crystal system	monoclinic	triclinic	monoclinic	monoclinic	orthorhombic
Space group	*P*2_1_/*c*	*P*$\bar{1}$	*P*2_1_/*c*	*P*2_1_/*n*	*Pbcn*
a/Å	8.0672(16)	7.5655(3)	11.2611(5)	11.672(2)	27.413(6)
b/Å	10.496(2)	11.7552(4)	10.6182(4)	6.5976(13)	7.2092(14)
c/Å	11.234(2)	31.9329(11)	8.2766(3)	12.193(2)	6.6259(13)
α/°	90.00	79.435(3)	90.00	90.00	90.00
β/°	109.66(3)	87.936(3)	95.406(3)	109.73(3)	90.00
γ/°	90.00	78.473(3)	90.00	90.00	90.00
Z	4	8	4	4	4
Volume/Å^3^	895.7(3)	2735.47(17)	985.26(7)	883.8(3)	1309.4(4)
ρ_calc_/g∙cm^−3^	1.692	1.832	1.707	1.617	1.782
μ/mm^−1^	0.154	0.178	0.152	0.137	0.160
F(000)	464	1520	512	448	728
Crystal size/mm^3^	0.13 × 0.12 × 0.08	0.50 × 0.15 × 0.14	0.40 × 0.35 × 0.30	0.15 × 0.13 × 0.10	0.16 × 0.14 × 0.03
θ range/°	3.31–27.49	3.04–26.00	3.13–26.00	2.97–27.50	2.92–27.48
Index ranges	−10 ≤ h ≤ 10; −13 ≤ k ≤ 13; −13 ≤ l ≤ 14	−9 ≤ h ≤ 9; −14 ≤ k ≤ 14; −32 ≤ l ≤ 39	−13 ≤ h ≤ 9; −13 ≤ k ≤ 12; −10 ≤ l ≤ 10	−14 ≤ h ≤ 15; −8 ≤ k ≤ 8; −15 ≤ l ≤ 15	−35 ≤ h ≤ 35; −9 ≤ k ≤ 6; −7 ≤ l ≤ 8
Reflections collected	6328	24085	4005	5836	5938
Independent reflections	2028	10754	1927	2007	1498
Goodness-of-fit on F^2^	1.203	1.077	1.073	1.122	1.157
Final R indexes [*I > 2σ(I)* i.e., *Fo > 4σ(Fo)*]	R1 = 0.0556; wR2 = 0.1121	R1 = 0.0552; wR2 = 0.1422	R1 = 0.0339; wR2 = 0.0785	R1 = 0.0456; wR2 = 0.1158	R1 = 0.0865; wR2 = 0.2420
Final R indexes [all data]	R1 = 0.0633; wR2 = 0.1171	R1 = 0.0607; wR2 = 0.1481	R1 = 0.0397; wR2 = 0.0820	R1 = 0.0477; wR2 = 0.1175	R1 = 0.0920; wR2 = 0.2500

**Table 2:** Physical properties of trinitromethyl derivatives of pyridine.

Compd	T_m_^a^	T_d_^b^	ρ ^c^	△_f_H_M_^d^	D ^e^	P ^f^	IS ^g^	FS ^h^	OB ^i^
**1**	70	114	1.62/1.69 ^j^	160.65	7678	24.2	16	>360	−56.11
**2**	96	101	1.77/1.83 ^j^	258.96	8700	33.2	9	192	−14.85
**3**	68	106	1.62/1.71 ^j^	314.22	7683	23.9	27	>360	−60.04
**4**	-	181	1.56/1.62 ^j^	151.32	7438	19.6	14	>360	−92.94
**5**	-	125	1.71/1.78 ^j^	150.80	8383	27.7	11.5	>360	−56.94
TNPyO ^k^	170	170	1.86	-	8369	-	1.5–3.0	160	−27.8
RDX ^l^	204	230	1.80	92.6	8795	34.9	7.4	120	−21.62
TNT ^l^	81	295	1.65	−59.4	7303	21.3	15	>353	−74.0

^a^ Melting point (onset) (°C); ^b^ Decomposition temperature (onset) (°C); ^c^ Density measured by a gas pycnometer at 25 °C (g·cm^−3^); ^d^ Calculated enthalpy of formation (KJ·mol^−1^); ^e^ Detonation velocity (m·s^−1^); ^f^ Detonation pressure (GPa); ^g^ Impact sensitivity (J); ^h^ Friction sensitivity (N); ^i^ Oxygen balance assuming the formation of CO_2_ at combustion (for C_a_H_b_O_c_N_d_, OB = 1600 (c − 2a − b/2)/MW, MW = molecular weight of compound) (%); ^j^ Crystal density at 298 K, recalculated from low-temperature X-ray densities, ρ_298K_ = ρ_T_/(1 + α_v_(298 − T_0_)) α_v_ = 1.5 × 10^−4^, T_0_ is the crystal testing temperature (g·cm^−3^); ^k^ Ref. [37]; ^l^ Ref. [38].

## Supplementary Materials

Supplementary materials can be accessed online.
