# Supplementary material for: Energetic Di- and Trinitromethylpyridines: Synthesis and Characterization
Source: Molecules. 2017 Dec 21;23(1):2. doi: 10.3390/molecules23010002 (PMC5943950; doi:10.3390/molecules23010002)
Supplement: Supplementary file 1 [file molecules-23-00002-s001.pdf]

Supplemantary Materials

Mono and di(trinitromethyl)-substituted pyridines and their salts: syntheses, characterization and energetic properties†

Table of Contents

1. ^{1}H and ^{13}C NMR spectral data

2. TG and DSC data

3. X-ray crystallography

4. Heat of formation calculations

^{1}H and ^{13}C NMR spectra

2-trinitromethylpyridine 1

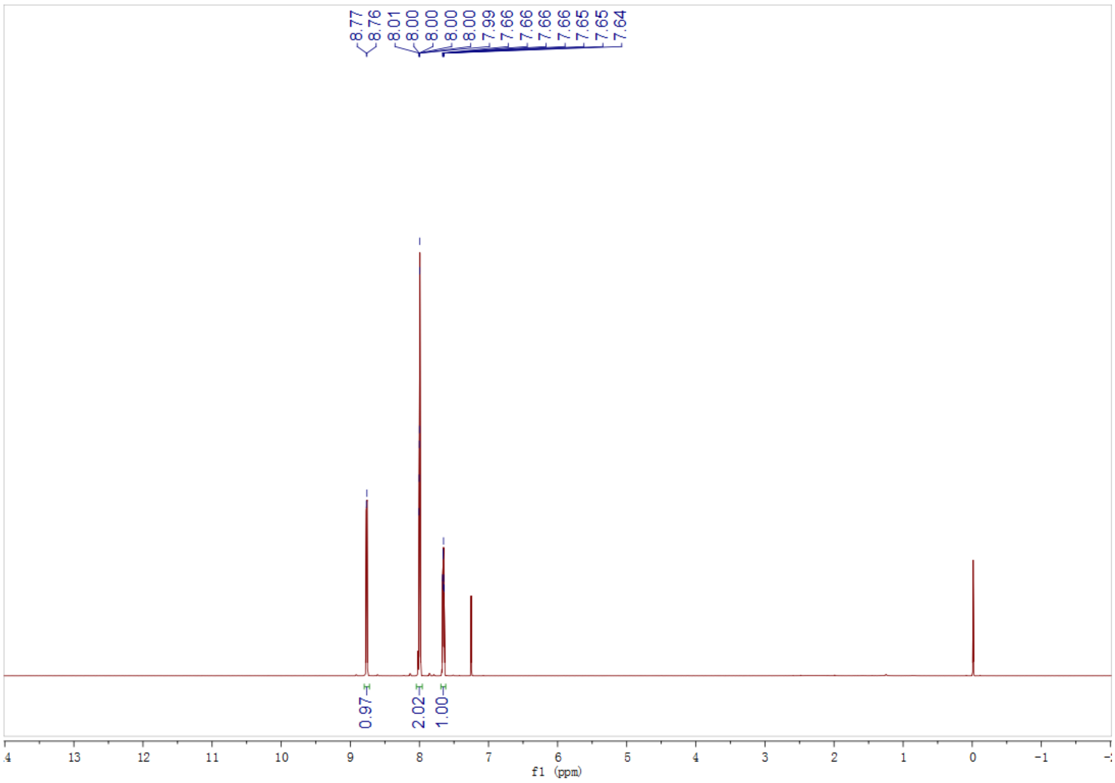

Figure S1. ^{1}H NMR spectrum of **1** in Chloroform-d

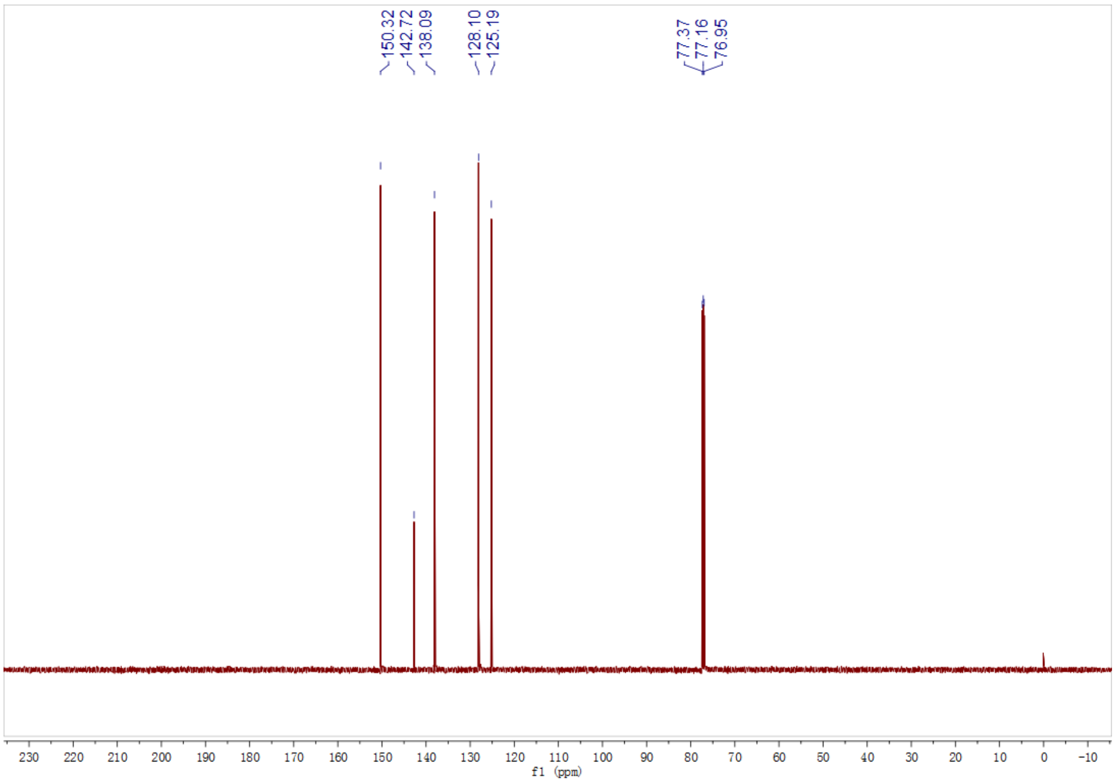

Figure S2. ^{13}C NMR spectrum of **1** in Chloroform-d

2,6-ditrinitromethylpyridine 2

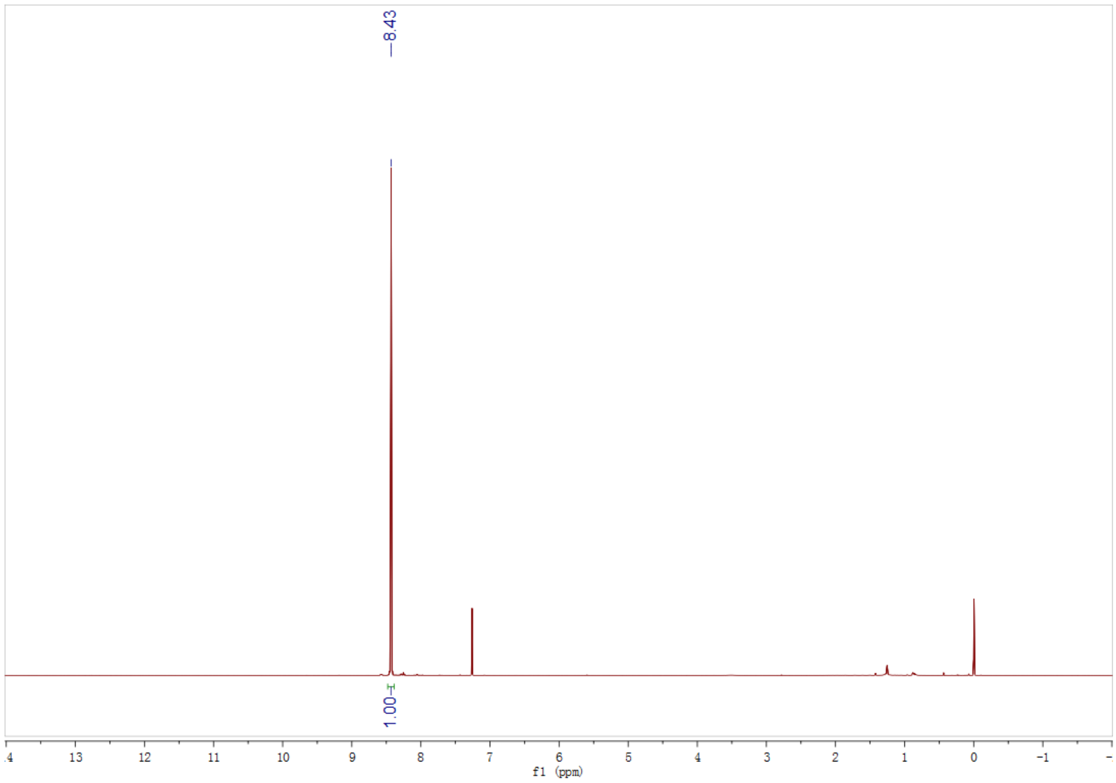

Figure S3. ^{1}H NMR spectrum of **2** in Chloroform-d

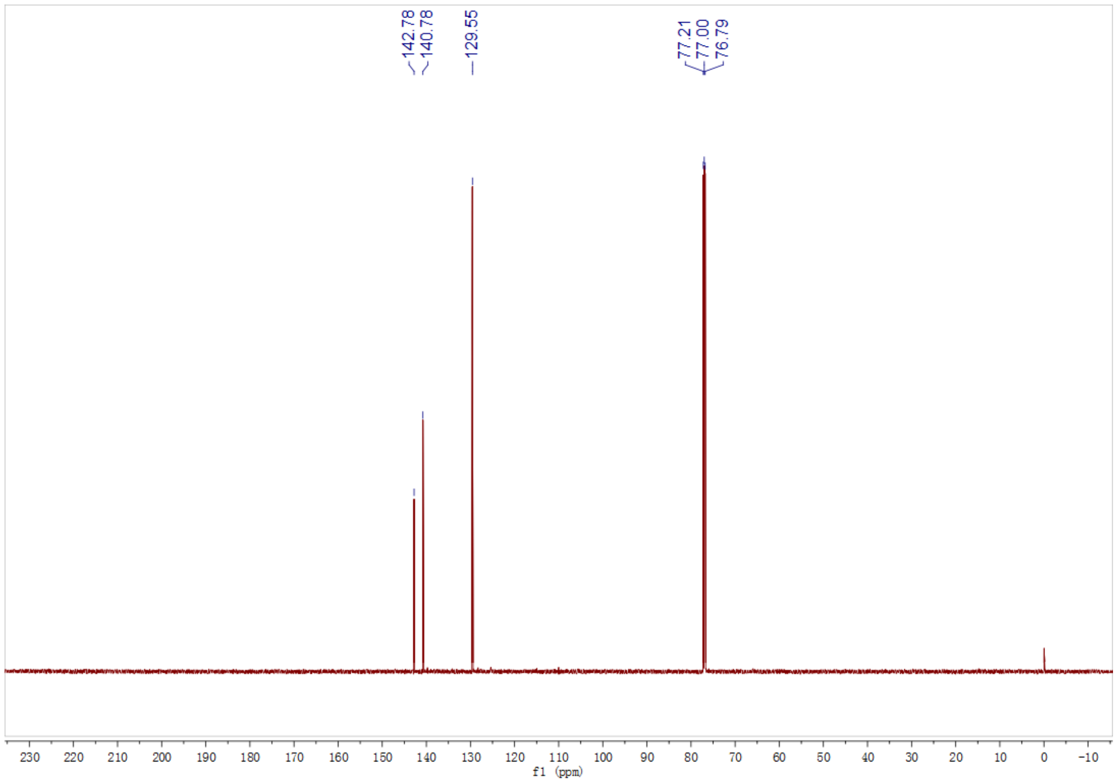

Figure S4. ^{13}C NMR spectrum of **2** in Chloroform-d

2-cyano-6-trinitromethylpyridine 3

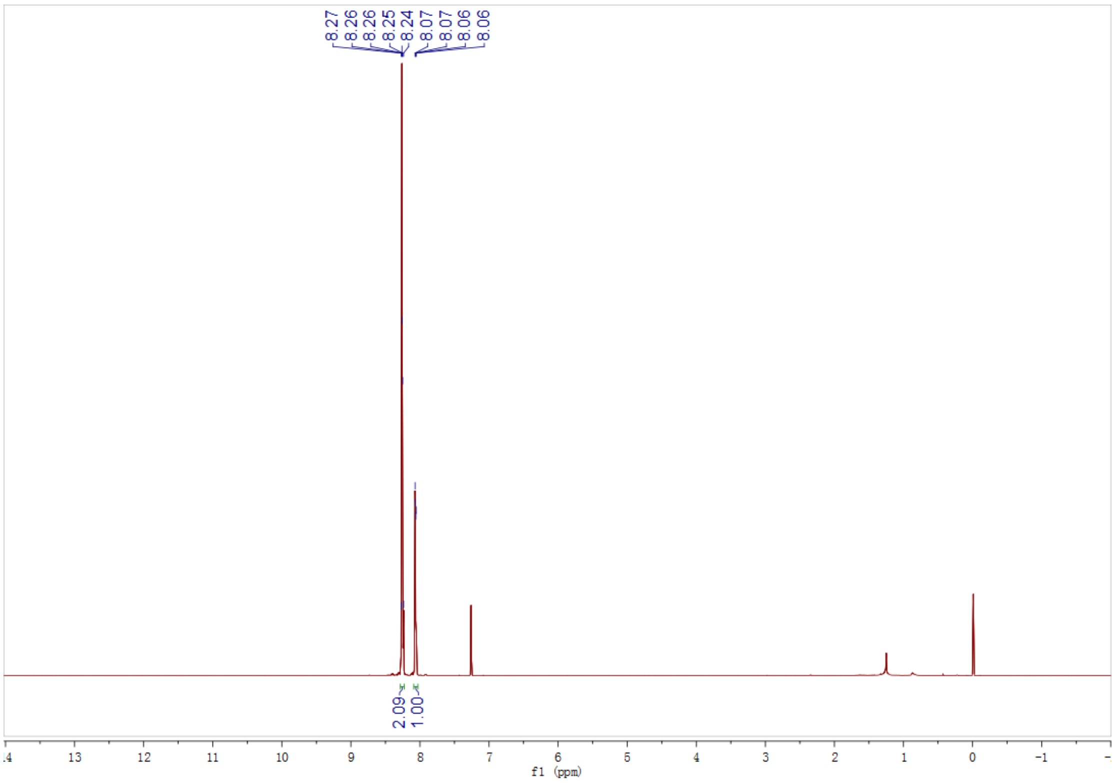

Figure S5. ^{1}H NMR spectrum of **3** in Chloroform-d

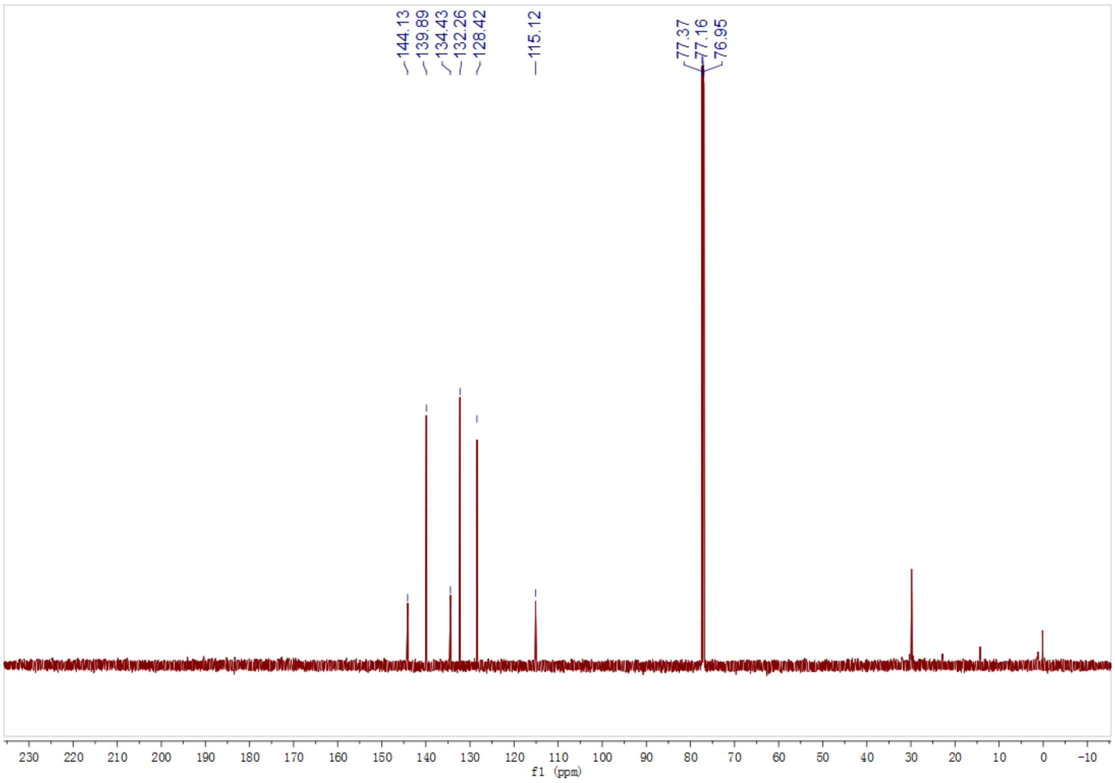

Figure S6. ^{13}C NMR spectrum of **3** in Chloroform-d

Hydrazinium 2-dinitromethylpyridine 4

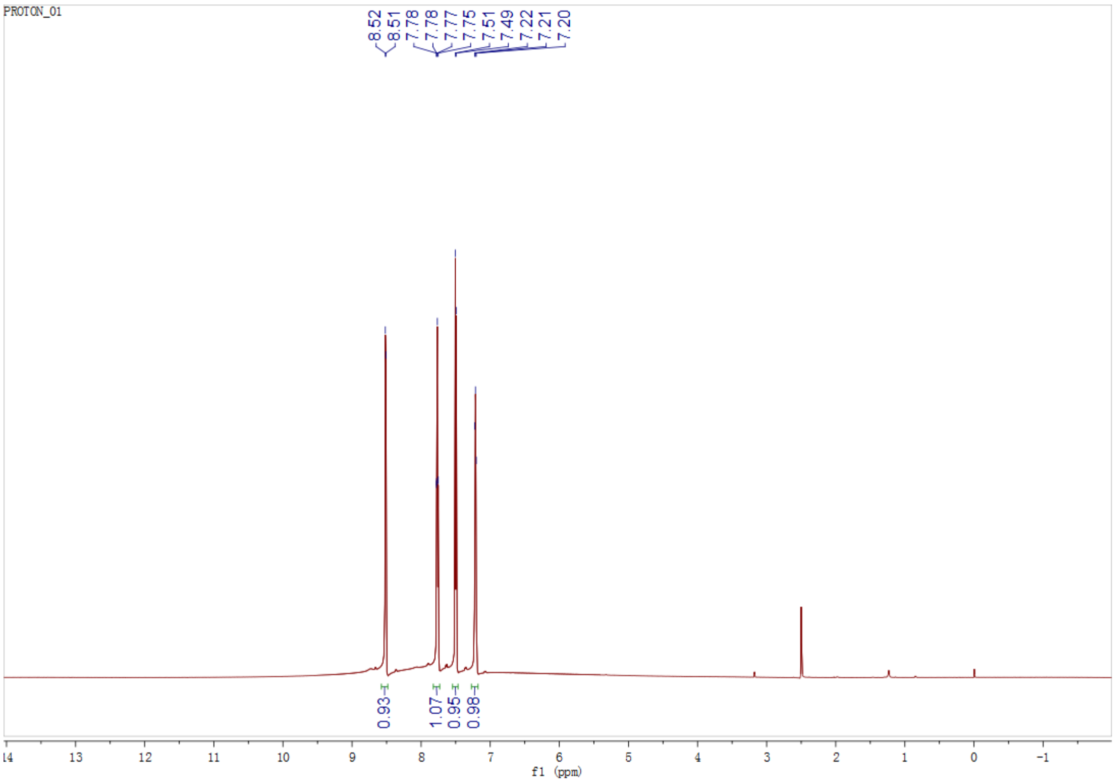

Figure S7. ^{1}H NMR spectrum of **4** in DMSO-d6

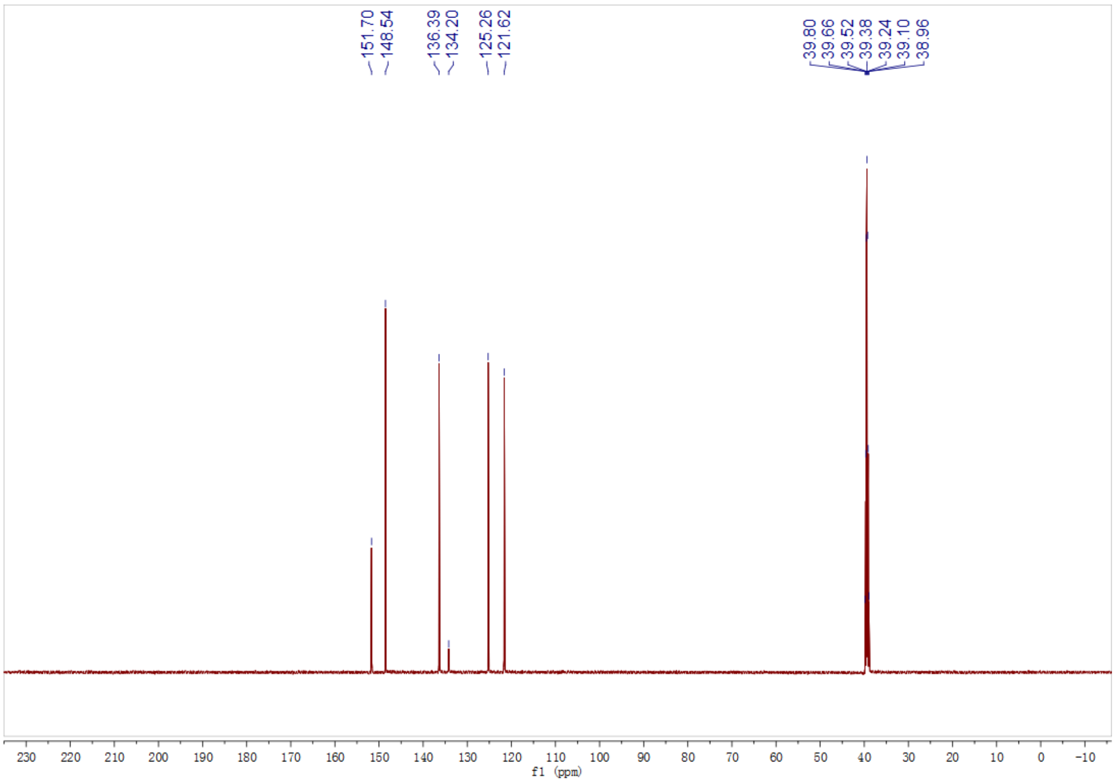

Figure S8. ^{13}C NMR spectrum of **4 i**n DMSO-d6

Dihydrazinium 2,6-di(dinitromethyl)pyridine 5

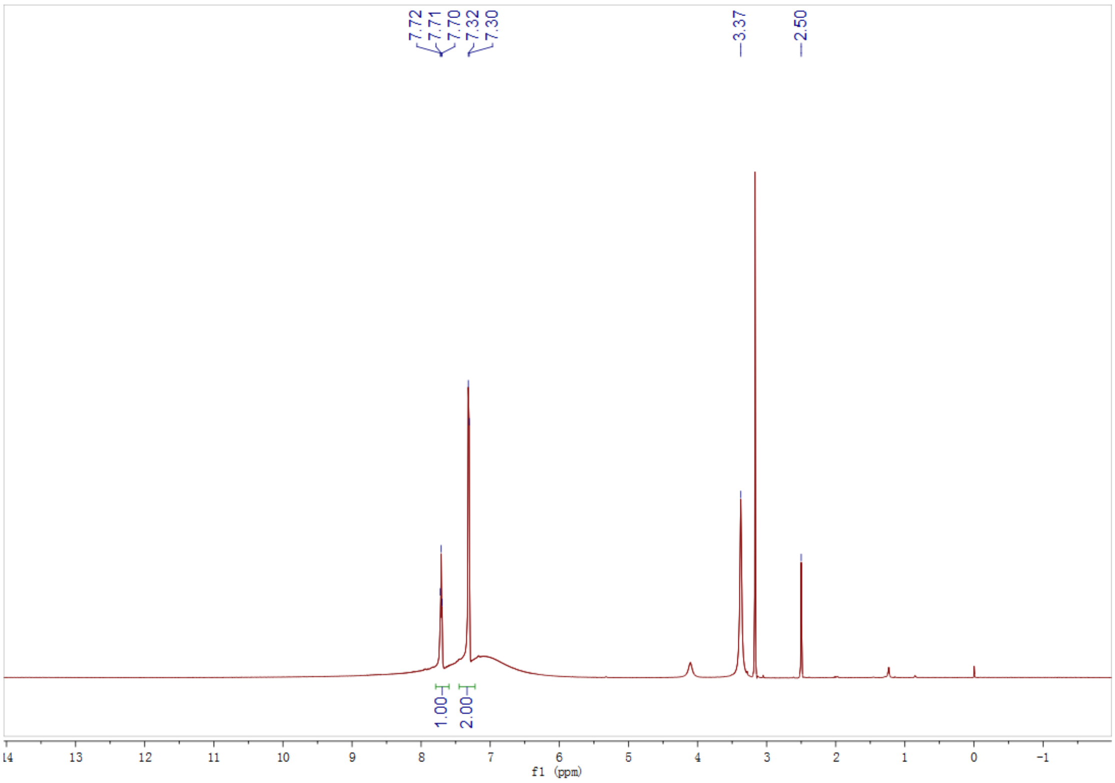

Figure S9. ^{1}H NMR spectrum of **5** in DMSO-d6

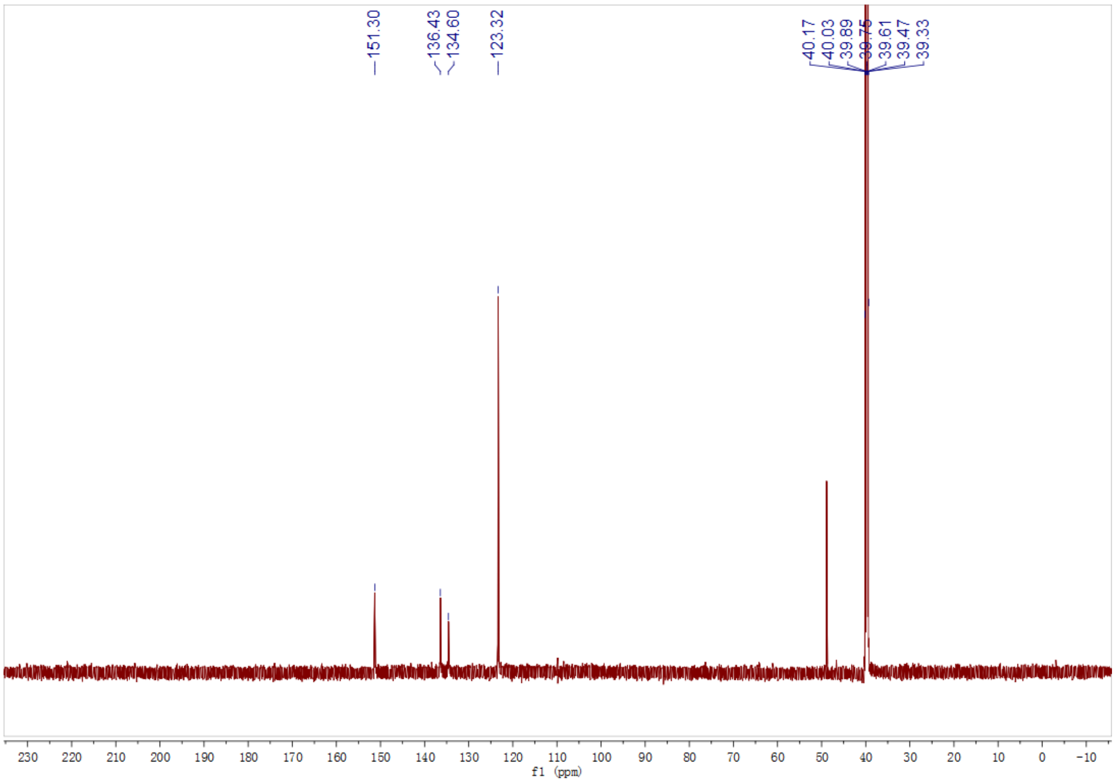

Figure S10. ^{13}C NMR spectrum of **5** in DMSO-d6

TG and DSC data

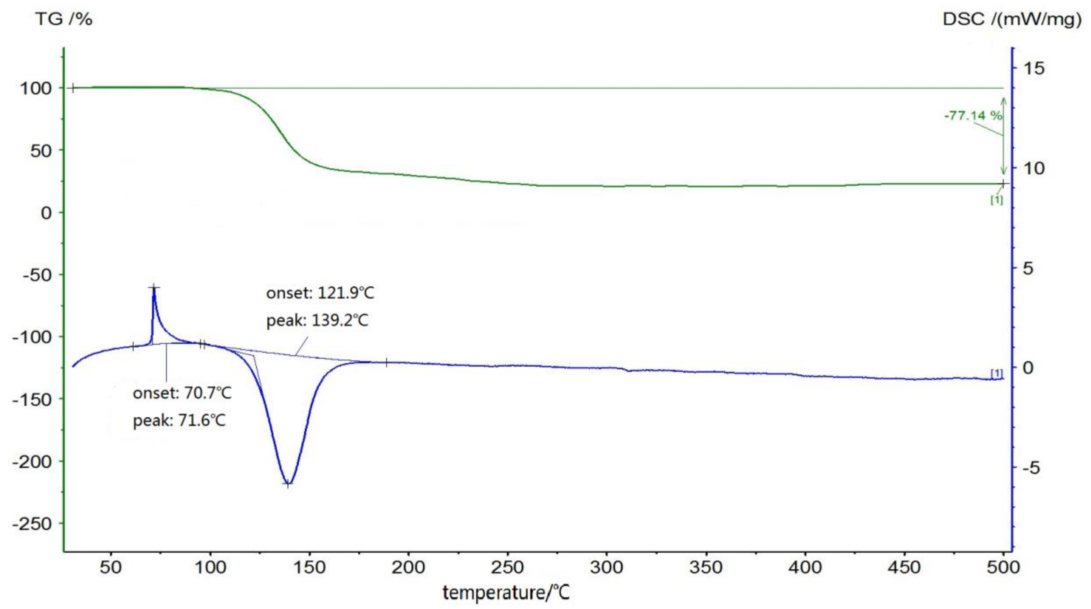

Figure S11. TG and DSC curves of **1**

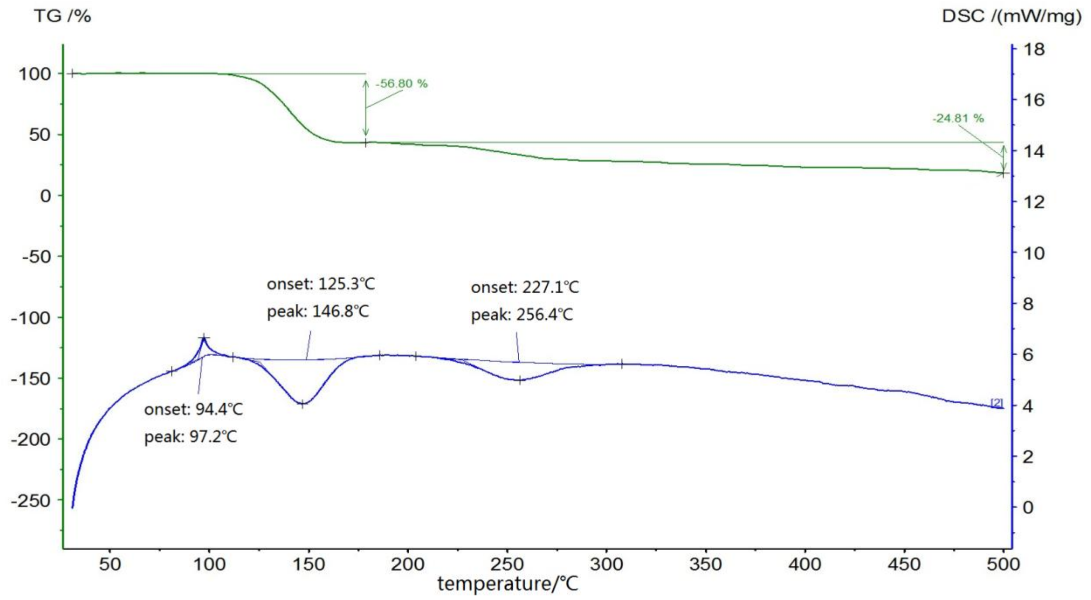

Figure S12. TG and DSC curves of **2**

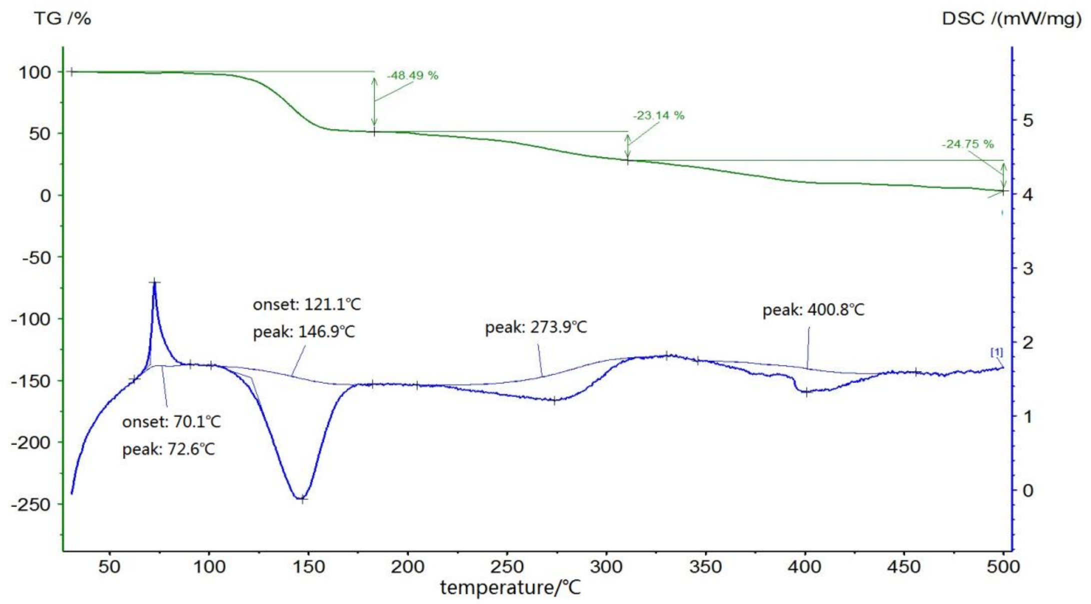

Figure S13. TG and DSC curves of **3**

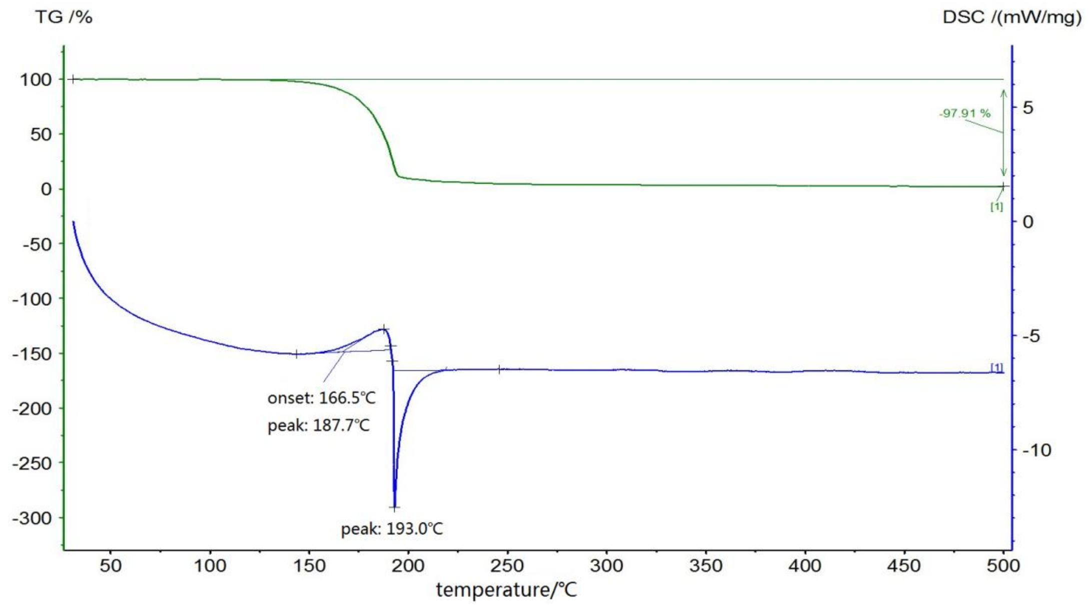

Figure S14. TG and DSC curves of **4**

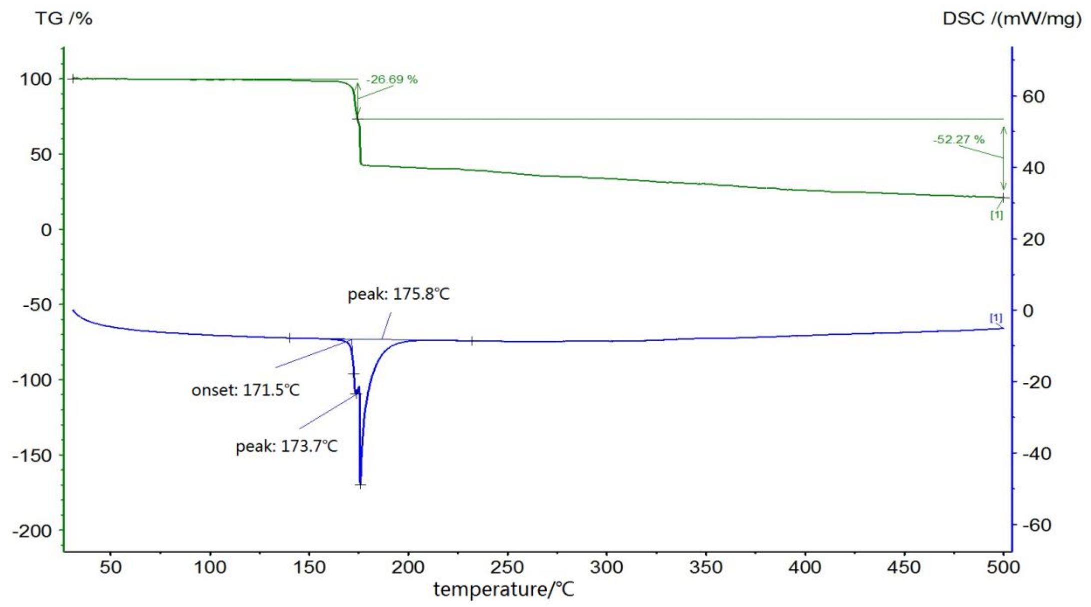

Figure S15. TG and DSC curves of **5**

X-ray crystallography

Table S1. Selected bond lengths [Å] and angles [°] for compound **1**

O1- N2	1.216(2)	O4 -N3 -C6	115.90(17)
O2 -N2	1.216(2)	O5 -N4 -O6	126.52(18)
O3 -N3	1.205(2)	O5 -N4 -C6	119.51(17)
O4 -N3	1.213(2)	O6 -N4 -C6	113.96(16)
O5 -N4	1.208(2)	N1 -C1 -H1	118.4
O6 -N4	1.220(2)	N1 -C1 -C2	123.12(19)
N1- C1	1.337(2)	C2 -C1 -H1	118.4
N1 -C5	1.341(2)	C1 -C2- H2	120.6
N2 -C6	1.528(3)	C3 -C2 -C1	118.83(18)
N3 -C6	1.538(2)	C3 -C2 -H2	120.6
N4 -C6	1.524(2)	C2 -C3 -H3	120.4
C1 -H1	0.9500 .	C2 -C3 -C4	119.18(18)
C1 -C2	1.388(3)	C4 -C3 -H3	120.4
C2 -H2	0.9500 .	C3 -C4 -H4	121.2
C2 -C3	1.374(3)	C5 -C4 -C3	117.64(18)
C3- H3	0.9500 .	C5- C4 -H4	121.2
C3 -C4	1.390(3)	N1 -C5- C4	124.49(17)
C4 -H4	0.9500 .	N1- C5 -C6	111.94(15)
C4 -C5	1.375(3)	C4 -C5 -C6	123.55(16)
C5 -C6	1.515(2)	N2 -C6 -N3	105.82(14)
C1- N1 -C5	116.71(16)	N4 -C6 -N2	108.47(15)
O1 -N2 -C6	115.28(15)	N4 -C6 -N3	104.14(14)
O2 -N2 -O1	126.98(19)	C5 -C6- N2	109.26(14)
O2 -N2 -C6	117.72(17)	C5 -C6 -N3	113.50(15)
O3 -N3 -O4	127.00(18)	C5 -C6 -N4	115.12(14)
O3 -N3 -C6	117.08(16)		

Table S2. Torsion angles for **1** [°]

O1- N2 -C6 -N3	-177.16(15)	O6- N4 -C6- N3	-60.2(2)
O1 -N2- C6 -N4	-65.92(19)	O6 -N4 -C6 -C5	64.7(2)
O1 -N2 -C6 -C5	60.3(2)	N1 -C1 -C2 -C3	-1.8(3)
O2 -N2 -C6 -N3	4.3(2)	N1 -C5 -C6 -N2	31.3(2)
O2 -N2 -C6 -N4	115.53(18)	N1 -C5 -C6 -N3	-86.55(19)
O2 -N2 -C6 -C5	-118.25(18)	N1 -C5 -C6 -N4	153.58(15)
O3 -N3 -C6 -N2	-100.4(2)	C1 -N1 -C5 -C4	0.9(3)
O3 -N3 -C6 -N4	145.4(2)	C1 -N1 -C5 -C6	179.47(16)
O3 -N3 -C6 -C5	19.4(3)	C1 -C2 -C3 -C4	1.4(3)
O4 -N3 -C6 -N2	77.9(2)	C2 -C3 -C4 -C5	0.1(3)
O4 -N3 -C6 -N4	-36.4(2)	C3 -C4 -C5 -N1	-1.3(3)
O4- N3- C6 -C5	-162.29(19)	C3 -C4 -C5- C6	-179.68(17)
O5- N4 -C6 -N2	6.5(2)	C4 -C5 -C6 -N2	-150.15(17)

O5- N4 -C6 -N3	118.86(18)	C4 -C5 -C6 -N3	92.0(2)
O5- N4 -C6 -C5	-116.24(19)	C4 -C5 -C6 -N4	-27.8(2)
O6 -N4 -C6 -N2	-172.59(15)	C5 -N1- C1 -C2	0.7(3)

Table S3. Selected bond lengths [Å] and angles [°] for compound **2**

O4 -N2	1.210(4)	O6 -N3 -C1	115.3(3)
O11- N7	1.243(5)	O8 -N5 -O7	126.6(3)
O12 -N7	1.175(5)	O8 -N5 -C7	116.3(3)
O10 -N6	1.222(4)	O7 -N5 -C7	117.1(3)
O9 -N6	1.221(4)	O1 -N1 -C1	114.5(3)
O8 -N5	1.213(4)	O2 -N1 -O1	128.1(3)
O3 -N2	1.227(4)	O2 -N1 -C1	117.3(3)
O7 -N5	1.237(4)	O4 -N2 -O3	128.3(3)
O5 -N3	1.215(5)	O4 -N2 -C1	115.8(3)
O6 -N3	1.222(4)	O3 -N2- C1	115.9(3)
O1 -N1	1.223(4)	N6 -C7 -N7	105.3(3)
O2 -N1	1.204(4)	N5 -C7 -N7	106.7(3)
N4 -C6	1.322(5)	N5 -C7 -N6	105.6(3)
N4 -C2	1.341(5)	C6 -C7 -N7	111.8(3)
N7 -C7	1.552(5)	C6 -C7 -N6	115.2(3)
N6 -C7	1.548(5)	C6 -C7 -N5	111.6(3)
N3 -C1	1.527(5)	N4 -C6- C7	113.8(3)
N5 -C7	1.519(5)	N4 -C6 -C5	124.8(4)
N1 -C1	1.546(5)	C5-C6- C7	121.4(3)
N2 -C1	1.533(4)	C6-C5 -H5	121.5
C7 -C6	1.501(5)	C4 C5 -C6	117.0(4)
C6 -C5	1.404(5)	C4-C5 -H5	121.5
C5 -H5	0.95	N4 -C2 -C3	124.7(4)
C5 -C4	1.362(6)	N4- C2 -C1	114.7(3)
C2 -C3	1.392(5)	C3 -C2 -C1	120.6(3)
C2 -C1	1.524(5)	C5 -C4 -H4	119.6
C4 -H4	0.95	C5 -C4 -C3	120.8(4)
C4 -C3	1.380(5)	C3 -C4 -H4	119.6
C3 -H3	0.95	C2 -C3 -H3	121.6
C6 -N4- C2	115.7(3)	C4 -C3 -C2	116.9(4)
O11- N7 -C7	116.6(3)	C4 -C3 -H3	121.6
O12 -N7 -O11	127.4(3)	N3 -C1- N1	108.4(3)
O12 -N7 -C7	116.1(3)	N3- C1 -N2	108.3(3)
O10 -N6- C7	115.1(3)	N2 -C1 -N1	104.8(3)
O9 -N6 -O10	127.1(3)	C2 -C1- N3	111.3(3)
O9 -N6- C7	117.8(3)	C2 -C1 -N1	112.6(3)
O5 -N3- O6	128.6(4)	C2- C1- N2	111.2(3)
O5 -N3 -C1	116.1(3)		

Table S4. Torsion angles for **2** [°]

O4-N2-C1-N3	-83.6(4)	O6-N3-C1-C2	-101.8(4)
O4-N2-C1-N1	161.0(3)	O1-N1-C1-N3	157.2(3)
O4-N2-C1-C2	39.0(4)	O1-N1-C1-N2	-87.4(3)
O11-N7-C7-N6	-20.9(4)	O1-N1-C1-C2	33.6(4)
O11-N7-C7-N5	91.0(4)	O2-N1-C1-N3	-25.1(4)
O11-N7-C7-C6	-146.7(3)	O2-N1-C1-N2	90.3(4)
O12-N7-C7-N6	159.0(3)	O2-N1-C1-C2	-148.6(3)
O12-N7-C7-N5	-89.1(4)	N4-C6-C5-C4	-2.8(6)
O12-N7-C7-C6	33.2(5)	N4-C2-C3-C4	-1.4(6)
O10-N6-C7-N7	-92.8(3)	N4-C2-C1-N3	-15.7(4)
O10-N6-C7-N5	154.4(3)	N4-C2-C1-N1	106.2(4)
O10-N6-C7-C6	30.8(4)	N4-C2-C1-N2	-136.6(3)
O9-N6-C7-N7	86.3(4)	N7-C7-C6-N4	-123.2(3)
O9-N6-C7-N5	-26.4(4)	N7-C7-C6-C5	54.7(5)
O9-N6-C7-C6	-150.0(3)	N6-C7-C6-N4	116.7(3)
O8-N5-C7-N7	-164.8(3)	N6-C7-C6-C5	-65.4(5)
O8-N5-C7-N6	-53.2(4)	N5-C7-C6-N4	-3.7(4)
O8-N5-C7-C6	72.7(4)	N5-C7-C6-C5	174.2(4)
O3-N2-C1-N3	95.6(3)	C7-C6-C5-C4	179.6(3)
O3-N2-C1-N1	-19.9(4)	C6-N4-C2-C3	-0.3(5)
O3-N2-C1-C2	-141.8(3)	C6-N4-C2-C1	179.8(3)
O7-N5-C7-N7	18.2(4)	C6-C5-C4-C3	0.8(6)
O7-N5-C7-N6	129.8(3)	C5-C4-C3-C2	1.1(6)
O7-N5-C7-C6	-104.3(4)	C2-N4-C6-C7	-179.8(3)
O5-N3-C1-N1	-47.2(4)	C2-N4-C6−C5	2.4(5)
O5-N3-C1-N2	-160.4(3)	C3-C2-C1-N3	164.3(3)
O5-N3-C1-C2	77.1(4)	C3-C2-C1-N1	-73.8(4)
O6-N3-C1-N1	133.9(3)	C3-C2-C1-N2	43.5(5)
O6-N3-C1-N2	20.8(4)	C1-C2-C3-C4	178.5(3)

Table S5. Selected bond lengths [Å] and angles [°] for compound **3**

N2- C6	1.3307(18)	O3 -N4 -C7	117.82(12)
N2 -C2	1.3447(18)	O6 -N5 -C7	113.48(12)
N3 -O2	1.2188(16)	O5 -N5 -O6	127.94(13)
N3 -O1	1.2113(16)	O5 -N5 -C7	118.52(12)
N3 -C7	1.5376(18)	N2 -C6 -C5	125.14(13)
N4 -O4	1.2121(17)	N2 -C6 -C7	115.51(12)
N4 -O3	1.2134(16)	C5 -C6 -C7	119.29(13)
N4 -C7	1.5397(18)	C4 -C3 -H3	120.8
N5 -O6	1.2172(16)	C4 -C3 -C2	118.32(13)
N5 -O5	1.2081(17)	C2 -C3 -H3	120.8
N5 -C7	1.5275(18)	C6 -C5 -H5	121.1
C6 -C5	1.391(2)	C4 -C5 -C6	117.77(14)

C6 -C7	1.5193(19)	C4 -C5 -H5	121.1
C3 -H3	0.93	N1 -C1 -C2	179.60(17)
C3 -C4	1.384(2)	C3 -C4 -C5	118.94(14)
C3 -C2	1.390(2)	C3 -C4 -H4	120.5
C5 -H5	0.93	C5 -C4 -H4	120.5
C5 -C4	1.384(2)	N2 -C2 -C3	124.25(14)
C1 -N1	1.148(2)	N2 -C2 -C1	115.33(13)
C1 -C2	1.454(2)	C3 -C2 -C1	120.41(13)
C4 -H4	0.93	N3 -C7 -N4	105.68(10)
C6 -N2 -C2	115.57(12)	N5 -C7 -N3	106.23(10)
O2 -N3 -C7	116.27(12)	N5 -C7- N4	110.34(11)
O1 -N3 -O2	127.15(13)	C6 -C7 -N3	114.50(11)
O1 -N3 -C7	116.55(11)	C6 -C7 -N4	109.17(11)
O4 -N4 -O3	127.13(13)	C6 -C7 -N5	110.77(11)
O4 -N4- C7	115.01(12)		

Table S6. Torsion angles for **3** [°]

N2 -C6 -C5 -C4	0.5(2)	O4 -N4- C7 -C6	-55.04(16)
N2 -C6 -C7 -N3	-117.08(13)	O3 -N4 -C7 -N3	-0.93(16)
N2 -C6 -C7 -N4	124.71(12)	O3 -N4 -C7 -N5	-115.36(14)
N2 -C6 -C7 -N5	3.01(17)	O3 -N4 -C7 -C6	122.68(13)
O2 -N3- C7- N4	-74.86(14)	C6 -N2 -C2 -C3	0.7(2)
O2 -N3 -C7- N5	42.38(15)	C6 -N2 -C2- C1	-179.61(12)
O2 -N3 -C7 -C6	164.96(12)	C6 -C5 -C4 -C3	0.1(2)
O6 -N5 -C7 -N3	46.37(14)	C5 -C6 -C7 -N3	65.56(16)
O6 -N5 -C7 -N4	160.46(12)	C5 -C6 -C7 -N4	-52.65(17)
O6 -N5 -C7 -C6	-78.53(14)	C5 -C6 -C7 -N5	-174.35(12)
O1 -N3 -C7 -N4	103.07(13)	C4 -C3 -C2 -N2	-0.1(2)
O1 -N3 -C7 -N5	-139.68(12)	C4 -C3 -C2 -C1	-179.79(14)
O1 -N3 -C7 -C6	-17.10(17)	N1- C1- C2 -N2	118(23)
O5 -N5 -C7 -N3	-136.20(13)	N1 -C1 -C2 -C3	-63(23)
O5 -N5 -C7 -N4	-22.11(17)	C2 -N2 -C6 -C5	-0.9(2)
O5 -N5 -C7 -C6	98.90(14)	C2 -N2 -C6 -C7	-178.08(12)
O4 -N4 -C7 -N3	-178.65(12)	C2 -C3 -C4 -C5	-0.3(2)
O4 -N4 -C7 -N5	66.91(15)	C7 -C6 -C5- C4	177.60(13)

Table S7. Selected bond lengths [Å] and angles [°] for compound 4

O1 -N1	1.2709(16)	C2 -N3- C6	117.25(13)
O2 -N1	1.2560(15)	N1 -C1 -N2	121.60(12)
O3 -N2	1.2441(16)	N1- C1 -C2	120.04(12)
O4 -N2	1.2538(17)	N2- C1 -C2	118.35(12)
N1 -C1	1.3618(18)	N3- C2 -C1	116.94(12)
N2 -C1	1.3907(18)	N3- C2 -C3	123.14(13)
N3 -C2	1.3368(19)	C3 -C2 -C1	119.93(13)

N3 -C6	1.3405(19)	C2 -C3 -H3	120.8
C1 -C2	1.4837(18)	C4 -C3 -C2	118.39(14)
C2 -C3	1.3940(19)	C4 -C3 -H3	120.8
C3 -H3	0.95	C3 -C4 -H4	120.4
C3 -C4	1.387(2)	C5 -C4 -C3	119.15(14)
C4 -H4	0.95	C5 -C4 -H4	120.4
C4 -C5	1.379(2)	C4 -C5 -H5	120.8
C5 -H5	0.95	C4 -C5 -C6	118.34(14)
C5 -C6	1.385(2)	C6 -C5 -H5	120.8
C6 -H6	0.95	N3 -C6 -C5	123.73(14)
N4 -H4A	0.8499	N3 -C6 -H6	118.1
N4 -H4B	0.8501	C5 -C6 -H6	118.1
N4 -H4C	0.8499	H4A -N4 -H4B	112.1
N4 -N5	1.4428(16)	H4A -N4 -H4C	104.6
N5 -H5A	0.85	H4B -N4- H4C	111.3
N5 -H5B	0.85	N5 -N4 -H4A	110.3
O1 -N1 -C1	116.49(11)	N5 -N4 -H4B	113.2
O2 -N1 -O1	119.15(12)	N5 -N4 -H4C	104.8
O2 -N1 -C1	124.36(12)	N4 -N5 -H5A	109.8
O3 -N2 -O4	120.95(12)	N4 -N5 -H5B	109.8
O3 -N2 -C1	123.82(12)	H5A- N5- H5B	104.7
O4 -N2- C1	115.23(12)		

Table S8. Torsion angles for **4** [°]

O1 -N1- C1 -N2	-176.03(12)	N2- C1 -C2- N3	94.52(16)
O1 -N1- C1 -C2	2.99(19)	N2- C1 -C2 -C3	-85.52(17)
O2 -N1- C1 -N2	3.7(2)	N3 -C2 -C3 -C4	0.6(2)
O2 -N1- C1 -C2	-177.23(13)	C1 -C2 -C3 -C4	-179.34(13)
O3 -N2 -C1 -N1	-9.4(2)	C2 -N3 -C6 -C5	0.7(2)
O3 -N2 -C1 -C2	171.52(13)	C2 -C3 -C4 -C5	0.2(2)
O4 -N2 -C1 -N1	171.24(13)	C3 -C4 -C5 -C6	-0.6(2)
O4 -N2 -C1- C2	-7.80(19)	C4 -C5 -C6 -N3	0.1(2)
N1 -C1 -C2- N3	-84.53(17)	C6 -N3 -C2 -C1	178.91(13)
N1 -C1 -C2- C3	95.43(17)	C6 -N3- C2 -C3	-1.0(2)

Table S9. Selected bond lengths [Å] and angles [°] for compound 5

O3- N3	1.254(3)	O4 -N3- O3	119.9(3)
O1- N2	1.243(4)	O4- N3 -C10	123.2(3)
N1 -C8	1.358(3)	O1- N2 -C10	118.0(3)
N1 -C8	1.358(3)	O2 -N2 -O1	120.6(3)
N3 -O4	1.245(4)	O2 -N2 -C10	121.3(3)
N3 -C10	1.395(4)	C8 -C7 -H7	120.6
N2 -C10	1.398(4)	C9 -C7 -H7	120.6
N2 -O2	1.241(4)	C9 -C7 -C8	118.8(3)
C7 -H7	0.9500 .	N1- C8 -C7	117.4(3)

C7 -C8	1.399(4)	N1- C8 -C10	119.3(3)
C7 -C9	1.382(4)	C7 -C8 -C10	123.3(3)
C8 -C10	1.457(4)	C7 -C9 -C7	122.0(4)
C9 -C7	1.382(4)	C7 -C9 -H9	119
C9 -H9	0.95	C7 -C9 -H9	119
N4 -H4AB	0.86	N3 -C10 -N2	118.3(3)
N4 -H4AA	0.86	N3 -C10 -C8	121.9(3)
N4 -H4BC	0.86	N2 -C10 -C8	119.8(3)
N4 -H4BD	0.86	N5 -N4 -H4AB	108
N4 -H4CF	0.86	N5 -N4 -H4AA	110.6
N4 -H4CE	0.8599	N6 -N4 -H4BC	110.5
N4 -H4DG	0.8599	N6 -N4 -H4BD	109.1
N4 -H4DH	0.8601	N8 -N4 -H4CF	109.4
N4 -N5	1.375(15)	N8 -N4 -H4CE	108.5
N4 -N6	1.416(15)	N9 -N4 -H4DG	110.1
N4 -N8	1.442(17)	N9 -N4 -H4DH	108.5
N4 -N9	1.419(17)	N4 -N5 -H5A	110.8
N5 -H5A	0.8902	N4 -N5 -H5B	108.9
N5 -H5B	0.89	N4 -N5 -H5C	108.7
N5 -H5C	0.89	H5A- N5 -H5	109.5
N6 -H6A	0.89	H5A -N5 -H5	109.5
N6 -H6B	0.8901	H5B -N5- H5	109.5
N6 -H6C	0.8893	N4 -N6 -H6A	108.4
N8 -H8A	0.8899	N4 -N6 -H6B	110.2
N8 -H8B	0.8899	N4 -N6 -H6C	109.8
N8 -H8C	0.8902	H6A- N6 -H6	109.4
N9 -H9A	0.89	H6A -N6 -H6	109.5
N9 -H9B	0.8902	H6B -N6- H6	109.4
N9 -H9C	0.8899	N4 -N8 -H8A	109.1
C8 -N1- C8	125.3(4)	N4 -N8 -H8B	110.3
O3 -N3 -C10	116.8(2)	N4 -N8- H8C	109

Table S10. Torsion angles for **5** [°]

O3- N3- C10- N2	174.0(3)	C7 -C8 -C10- N2	-30.6(5)
O3 -N3 -C10 -C8	-8.4(4)	C8 -N1 -C8 -C7	1.61(19)
O1 -N2 -C10- N3	160.7(3)	C8 -N1- C8 -C10	-179.0(3
O1 -N2- C10- C8	-16.9(4)	C8- C7 -C9 -C7	1.6(2)
N1 -C8 -C10 -N3	-27.5(4)	C9 -C7 -C8 -N1	-3.2(4)
N1 -C8 -C10 -N2	150.0(3)	C9 -C7 -C8 -C10	177.4(2)
O4 -N3 -C10 -N2	-9.1(5)	O2- N2 -C10- N3	-20.8(5)
O4 -N3 -C10 -C8	168.5(3)	O2 -N2- C10 -C8	161.6(3)
C7 -C8- C10 -N3	151.8(3)		

Heat of formation calculations

The isodesmic reactions which are used to calculate the HOF of the target compounds are shown in Scheme S1.

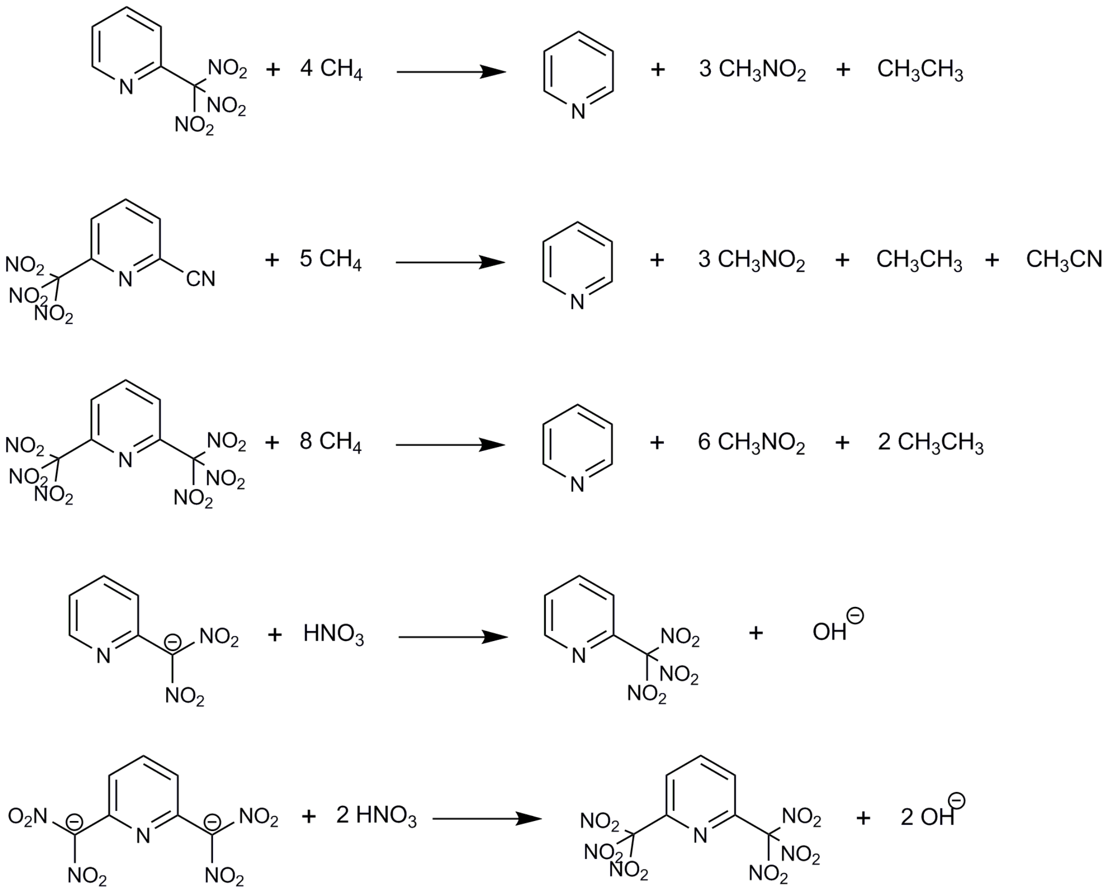

Scheme S1. Isodesmic reactions for 1, 2, 3, 4 anion and 5 anion.

The change of enthalpy for the reactions at 298 K can be expressed as Equation (1).

$$\Delta H_{298} = \Sigma\Delta_f H_P - \Sigma\Delta_f H_R \quad (1)$$

$\Delta_f H_R$ and $\Delta_f H_P$ are the HOF of the reactants and products at 298 K, respectively.

ΔH_{298} can also be calculated as follows:

$$\Delta H_{298} = \Delta E_{298} + \Delta(PV) = \Delta E_0 + \Delta ZPE + \Delta H_T + \Delta nRT \quad (2)$$

E_0 is the total energy; ZPE is the zero-point energies (ZPE); H_T is the thermal correction. On the right side of the Equation (2), it is the sum of the changes of corresponding parameters between the products and the reactants. For the isodesmic reactions, $\Delta n = 0$, so $\Delta(PV) = 0$. Combining Equation (1) and (2), the HOF of target compounds can be obtained.

Based on the Born-Haber energy cycle, the heats of formation of ionic salts can be simplified by Equation (3):

$$\triangle H_f^o(\text{ionic salts, 298 K}) = \triangle H_f^o(\text{cation, 298 K}) + \triangle H_f^o(\text{anion, 298 K}) - \triangle H_L \quad (3)$$

$\triangle H_L$ is the lattice energy of the ionic salts, which could be predicted by using the formula suggested by Jenkins et al.[1]

$$\triangle H_L = U_{POT} + [p(n_M/2 - 2) + q(n_X/2 - 2)]RT \quad (4)$$

n_M and n_X depend on the nature of the ions M^{p+} and X^{q-}, respectively, and are equal to three for monoatomic ions, five for linear polyatomic ions, and six for nonlinear polyatomic ions. The equation for lattice potential energy U_{POT} has the form Equation (5):

$$U_{POT}\ (\text{kJ mol}^{-1}) = \gamma(\rho_m/M_m)^{1/3} + \delta \quad (5)$$

ρ_m is the density (g cm^{-3}) and M_m is the chemical formula mass of the ionic material (g mol^{-1}), and the coefficients γ (kJ mol^{-1} cm) and δ (kJ mol^{-1}) are assigned literature values.

Table S11. Calculated total energy (E_0), zero-point energy (ZPE), thermal correction (HT), and heat of formation (HOF) of target compounds.

**Compd.**	**E_0/a.u.**	**ZPE (kJ/mol)**	**H_T (kJ/mol)**	**HOF (kJ/mol)**
**1**	-896.28	321.84	37.54	160.65
**2**	-1545.79	409.60	62.22	258.96
**3**	-988.02	317.07	42.27	314.22
**4 anion**	-692.26	283.55	31.51	-128.10
**5 anion**	-1137.69	336.06	44.63	-174.33
**$N_2H_5^+$**				774.1[2]

References

1. H. D. B. Jenkins, D. Tudeal and L. Glasser, L. *Inorg. Chem.*, 2002, **41**, 2364–2367.

2. D. Fischer, T. M. Klapçtke, M. Reymann and J. Stierstorfer, *Chem. Eur. J.*, 2014, **20**, 6401–6411.
